# RSK3 switches cell fate: from stress-induced senescence to malignant progression

**DOI:** 10.1186/s13046-023-02909-5

**Published:** 2023-11-27

**Authors:** Anda Huna, Jean-Michel Flaman, Catalina Lodillinsky, Kexin Zhu, Gabriela Makulyte, Victoria Pakulska, Yohann Coute, Clémence Ruisseaux, Pierre Saintigny, Hector Hernandez-Vargas, Pierre-Antoine Defossez, Mathieu Boissan, Nadine Martin, David Bernard

**Affiliations:** 1 Cancer Research Center of Lyon, Inserm U1052, CNRS UMR 5286, Université de Lyon, Centre Léon Bérard, 69373 Lyon, France; 2 Equipe Labellisée La Ligue Contre Le Cancer, Lyon, France; 3 INSERM UMR_S 938, Saint-Antoine Research Center, CRSA, University Sorbonne, Paris, France; 4 Research Area, Instituto de Oncología Ángel H. Roffo, Universidad de Buenos Aires, Consejo Nacional de Investigaciones Científicas y Técnicas (CONICET), Buenos Aires, Argentina; 5 Université Grenoble Alpes, Inserm, UA13 BGE, CNRS, CEA, FR2048, 38000 Grenoble, France; 6 Epigenetics and Cell Fate Centre, CNRS UMR 7216, Université Paris Diderot, Paris, France

**Keywords:** Cellular senescence, Epithelial-mesenchymal transition, TGFβ, Breast tumor

## Abstract

**Background:**

TGFβ induces several cell phenotypes including senescence, a stable cell cycle arrest accompanied by a secretory program, and epithelial-mesenchymal transition (EMT) in normal epithelial cells. During carcinogenesis cells lose the ability to undergo senescence in response to TGFβ but they maintain an EMT, which can contribute to tumor progression. Our aim was to identify mechanisms promoting TGFβ-induced senescence escape.

**Methods:**

In vitro experiments were performed with primary human mammary epithelial cells (HMEC) immortalized by hTert. For kinase library screen and modulation of gene expression retroviral transduction was used. To characterize gene expression, RNA microarray with GSEA analysis and RT-qPCR were used. For protein level and localization, Western blot and immunofluorescence were performed. For senescence characterization crystal violet assay, Senescence Associated-β-Galactosidase activity, EdU staining were conducted. To determine RSK3 partners FLAG-baited immunoprecipitation and mass spectrometry-based proteomic analyses were performed. Proteosome activity and proteasome enrichment assays were performed. To validate the role of RSK3 in human breast cancer, analysis of METABRIC database was performed. Murine intraductal xenografts using MCF10DCIS.com cells were carried out, with histological and immunofluorescence analysis of mouse tissue sections.

**Results:**

A screen with active kinases in HMECs upon TGFβ treatment identified that the serine threonine kinase RSK3, or RPS6KA2, a kinase mainly known to regulate cancer cell death including in breast cancer, reverted TGFβ-induced senescence. Interestingly, RSK3 expression decreased in response to TGFβ in a SMAD3-dependent manner, and its constitutive expression rescued SMAD3-induced senescence, indicating that a decrease in RSK3 itself contributes to TGFβ-induced senescence. Using transcriptomic analyses and affinity purification coupled to mass spectrometry-based proteomics, we unveiled that RSK3 regulates senescence by inhibiting the NF-κΒ pathway through the decrease in proteasome-mediated IκBα degradation. Strikingly, senescent TGFβ-treated HMECs display features of epithelial to mesenchymal transition (EMT) and during RSK3-induced senescence escaped HMECs conserve EMT features. Importantly, RSK3 expression is correlated with EMT and invasion, and inversely correlated with senescence and NF-κΒ in human claudin-low breast tumors and its expression enhances the formation of breast invasive tumors in the mouse mammary gland.

**Conclusions:**

We conclude that RSK3 switches cell fate from senescence to malignancy in response to TGFβ signaling.

**Supplementary Information:**

The online version contains supplementary material available at 10.1186/s13046-023-02909-5.

## Background

Cellular senescence is induced by stresses promoting tumor formation, for instance oncogenic activation, and it results in a stable cell cycle arrest, stopping tumor initiation, and malignant transformation [1, 2]. Cells undergoing senescence display an altered metabolism, morphology, redox regulation capacity, DNA damage, and have a complex senescence-associated secretory phenotype (SASP) [3, 4]. This SASP can reinforce senescence and can strongly impact the tissue microenvironment by secreting chemokines, cytokines, growth factors, matrix components, and/or metalloproteases that propagate senescence and provoke the elimination of potentially harmful cells [5, 6]. Nevertheless, emerging evidence shows that the accumulation of senescent cells, which naturally occurs during aging or after some chronic stresses, can promote long-term tumorigenesis, especially through their SASP, by promoting inflammation, fibrosis, and/or epithelial to mesenchymal transition (EMT) [7–10].

TGFβ (transforming growth factor beta), a pleiotropic signaling molecule, can be part of the SASP and can mediate some of its effects through the induction and reinforcement of senescence and/or by inducing EMT, among others [7, 11–17]. Hence, TGFβ seems to exert both anti-tumoral effects, by preventing uncontrolled cell proliferation during the initial steps of tumorigenesis, and pro-tumoral effects, by inducing EMT for instance, reminiscent of the effects of senescent cells.

To identify a potential pro-tumoral switch from anti- to pro-tumoral effects of such molecules, we screened a library of active kinases, which are often dysregulated in cancer [18]. We performed this screening on 192 active kinases to identify those promoting escape from TGFβ-induced senescence in normal human mammary epithelial cells (HMECs), which are able to both enter senescence arrest and EMT in response to TGFβ, and the mechanism(s) they control. Our data support that an underexplored kinase, RPSK6A2 (Ribosomal Protein S6 Kinase A2) also known as RSK3, inhibits TGFβ-induced senescence and promotes malignant progression of breast cancer.

## Methods

### Cell culture and treatment

HMEC from different donors were provided by Lonza and hTERT-immortalized HMECT generated within 2–3 passages of primary HMEC culture. HMEC and HMECT were grown in Mammary Epithelial Cell Growth Medium (MECGM, Promocell) supplemented with 1% penicillin/streptomycin (Life Technologies). 293GP (Clonotech) cells were grown in Dulbecco’s modified Eagle’s medium (DMEM, Life Technologies) supplemented with 10% fetal bovine serum (FBS) (Eurobio) and 1% penicillin/streptomycin. Cells were thawed, amplified, and frozen upon receipt. The experiments were performed on the aliquots within a month. Cells were maintained at 37 °C under a 5% CO_2_ atmosphere. TGFβ (PeproTech) was used at the concentration of 0.5 ng/mL. TNFα (PeproTech) was used at the concentration of 20 ng/mL.

### Retroviral transduction

The retroviral vectors used were pBABEhygro/hTERT (Addgene plasmid # 1773) [19], pWZLneo/RPS6KA2 (pWZL/RSK3) (Addgene plasmid # 20621) [20], pFLAG-CMV2/RSK3 [21], pBABE-puro-IκBα-mut super repressor (Addgene plasmid # 15291) [20], LPCX/SMAD2 (Addgene plasmid # 12636) [22], LPCX/SMAD3 (Addgene plasmid # 12638) [22], pBABE-puro-SMAD4 (Addgene plasmid # 37041) [23] together with their empty vector counterparts as controls. pWZL/RSK3 mutants were generated using the QuikChange II XL Site-Directed Mutagenesis Kit according to the manufacturer’s recommendations (Agilent Technologies), using the following primers for RSK3 K100R mutant: forward AGGGCTTTGAGAAGGCAGATC, reverse TCCAGGATCAGGTAGAGCTTTC or for RSK3 K464R: forward CAGTTACACGGGAACAACATC and reverse CATCAGCTCCATTACCAGGTAC and they were sequenced prior to usage (Genewiz). Virus-producing 293GP cells were transfected with vectors using GeneJuice reagent according to the manufacturer’s recommendations (Merck Millipore). Cells were transfected with VSVg (1 µg) and retroviral vector of interest (5 µg). Two days after transfection, the viral supernatant was mixed with fresh medium (1/2) and hexadimethrine bromide (8 μg/mL; Sigma‐Aldrich), and was then used to infect target cells for 6 h. One day after infection, selection started using puromycin at 500 ng/mL or/and neomycin at 100 μg/mL.

### Kinase library screen

The Myristoylated Kinase Library (Addgene) was used. All plasmids were prepared and used in separate wells. HMECT were seeded in 96-well plates at 2 × 10^3^ cells/well and infected 24 h after, one kinase per well. Twenty-four hours later cells were treated with TGFβ. 72 h after treatment cells were fixed with 3.7% formaldehyde and stained with Hoechst 33342 nucleic acid stain. Plates were read with Cytell cell imaging system (GE Healthcare Life Sciences) and then ranked by nuclei count.

### Crystal violet assay

The cells were plated at a density of 1 × 10^4^ cells/well in 6-well plates. Seven to ten days after treatment, the cells were rinsed with PBS, fixed with paraformaldehyde 3.7% for 15 min, washed and stained with Crystal Violet Solution 0.05%.

### Senescence associated-β-galactosidase activity

SA-β-Gal activity was carried out in 6-well plates at a density of 1 × 10^4^ cells/well. To measure the activity, cells were rinsed with PBS and fixed with Fixation Solution β-Gal (Glutaraldehyde 0.5%) for 5 min and incubated with the SA-β-Gal solution (40 mM citric acid, 5 mM K4[Fe(CN)6] 3H_2_O, 5 mM K3[Fe(CN)6, 150 mM NaCl, 2 mM MgCl_2_ 6H_2_O, X-gal (20 mg/mL)) overnight at 37 °C. At least 100 cells were counted for each condition.

### EdU staining

Proliferation assay was performed in 96-well plates with 1,500 cells/well. Cells were stained according to the manufacturer’s protocol with Click-iT Reaction Cocktail (Thermo Fischer Scientific). Wells were imaged with an Operetta High-Content Imaging System (PerkinElmer) and analyzed with Columbus software (PerkinElmer), to calculate the number of cells and the percentage of proliferating cells.

### RNA extraction, reverse transcription and quantitative PCR

NucleoZol (Macherey–Nagel) was used to perform phenol–chloroform extraction of RNA, and the RNA concentration was measured using NanoDropTM One/OneC (Ozyme). The Maxima First Strand cDNA Synthesis Kit (Fischer Scientific) was used for cDNA synthesis from 1 µg RNA. The RT mixture was diluted 1/10 and used for qPCR analysis. TaqMan mix or ONEGreen® Fast qPCR Premix (Ozyme) was used to perform qPCR with an FX96 Thermocycler (Bio-Rad). All reactions were performed in duplicate. The amount of mRNA was calculated with Ct (ΔΔCT) method and normalized against GAPDH. Primers (intron spanning) are listed in Supplemental Table 1.

### Transcriptome and GSEA analysis

Transcriptome analysis was performed using the Human GE 4 × 44 K v2 Microarray (Agilent Technologies) and the one-color gene expression Agilent workflow. Briefly, total RNA was extracted from HMECTs with the Nucleospin RNA kit (Macherey–Nagel) and RNA quality was checked using Tapestation (Agilent Technologies). cRNAs were then synthesized and labeled with Cy3 dye from 100 ng of total RNA using the one-color Low Input Quick Amp Labeling Kit (Agilent Technologies). After labeling and quality control validation, 1650 ng of Cy3-labeled cRNAs were hybridized to the 4 × 44 K arrays for 17 h at 65 °C. Microarrays were washed and scanned with an Agilent DNA microarray scanner G2565CA (Agilent Technologies). Fluorescent signals were extracted using Feature Extraction Software (version 10.5.1.1; Agilent Technologies) and transferred to Genespring GX 12.6 software (Agilent Technologies) for data processing and data mining. Data were normalized in Genespring using the 75^th^ percentile method. Microarray probes were filtered using the Agilent flag filter to remove probes with a raw signal below 10 in all replicates and for all conditions tested. For each condition, three independent replicates were analyzed and the average log2 fold change for all of the genes were determined in tested conditions compared to control conditions. Datasets are available in GEO GSE243320.

For GSEA analysis, all genes were ranked by their log2 fold-change expression according to the microarray data, in a condition of interest compared to its control condition. These ranked expression files were used for Gene Set Enrichment Analysis (GSEA) using the pre-ranked method of the GSEA v2.0.13 software with default parameters. Gene sets were obtained from the GSEA Molecular Signatures Database (MSigDB) (www.broadinstitute.org/gsea/).

### Western blot

Proteins were extracted with 2X Laemmli buffer and separated by SDS-PAGE in Tris–HCl-Glycine-SDS TGS pH 8.5 (Euromedex). Proteins were transferred onto a nitrocellulose membrane (BioRad) in Tris–Glycine pH 8.5 Buffer (Euromedex). The membranes were blocked for 1 h with milk 5% TBS-T 0.05%, and then incubated overnight at 4 °C with primary antibodies (Supplemental Table 2) in TBS-T milk 2.5%. Membranes were incubated with secondary antibodies (Supplemental Table 2) for 1 h at room temperature in TBS-T milk 2.5%. Pierce ECL Western blotting substrate (Life Technologies) or Clarity Max ECL substrate kit (Bio-Rad) was used for signal detection.

### Immunofluorescence

Cells were plated at 1.5 × 10^3^ cells/well in Cell Carrier 96-well plates (PerkinElmer), fixed with 3.7% formaldehyde 5 days after treatment with TGFβ, then permeabilized with Triton, and blocked with 20% FBS. The plates were incubated with the primary antibodies (Supplemental Table 2) overnight at 4 °C. The plates were rinsed three times with PBS-Tween and incubated with secondary antibodies (Supplemental Table 2) for 1 h at room temperature in the dark. Finally, the plates were co-stained with Hoechst (1/1,000 in PBS) for 10 min in the dark. Images were acquired with Operetta and analyzed using Columbus software (PerkinElmer).

### Immunoprecipitation

293GP cells were transfected with pFLAG-CMV2 or pFLAG-CMV2/RSK3, and 48 h later collected for immunoprecipitation. 2 × 10^8^ 293GP cells were extracted with lysis buffer (50 mM Tris pH 8.0, 0.5% NP-40, 200 mM NaCl, 0.1 mM EDTA, 10% glycerol), and protease inhibitors (Complete EDTA-free; Roche), quantified using the Protein Quantification Assay (Macherey–Nagel) and incubated overnight at 4 °C on the wheel with Anti-FLAG M2 affinity gel (Sigma). Samples were eluted with 3XFlag peptide (Sigma) or Laemmli buffer and visualized by Western blotting.

### Mass spectrometry-based proteomic analyses

Proteins eluted from the co-immunoprecipitation experiments (three replicates from FLAG-RSK3 expressing cells and three replicates from negative control cells) were solubilized in Laemmli buffer and stacked at the top of a 4–12% NuPAGE gel (Invitrogen). After staining with R-250 Coomassie Blue (Bio-Rad), the proteins were digested in-gel using trypsin (modified, sequencing purity, Promega), as previously described [24]. The resulting peptides were analyzed by online nanoliquid chromatography coupled with MS/MS (Ultimate 3000 RSLCnano and Q-Exactive Plus, Thermo Fisher Scientific) using a 90 min gradient. For this purpose, the peptides were sampled on a pre-column (300 μm × 5 mm PepMap C18, Thermo Scientific) and separated in a 75 μm × 250 mm C18 column (Reprosil-Pur 120 C18-AQ, 1.9 μm, Dr. Maisch). The MS and MS/MS data were acquired using Xcalibur (Thermo Fisher Scientific).

Peptides and proteins were identified by Mascot (version 2.8.0, Matrix Science) through concomitant searches against the Uniprot database (Homo sapiens taxonomy, 20210624 download) and a homemade database containing the sequences of classical contaminant proteins found in proteomic analyses (bovine albumin, keratins, trypsin, etc.). Trypsin/P was chosen as the enzyme and two missed cleavages were allowed. Precursor and fragment mass error tolerances were set at respectively at 10 and 20 ppm. Peptide modifications allowed during the search were: Carbamidomethyl (C, fixed), Acetyl (Protein N-term, variable) and Oxidation (M, variable). The Proline software [25] was used for compilation, grouping, and filtering of the results (conservation of rank 1 peptides, peptide length ≥ 6 amino acids, false discovery rate of peptide-spectrum-match identifications < 1% ^65^, and minimum of one specific peptide per identified protein group). Proline was then used to perform a MS1 quantification of the identified protein groups based on razor and specific peptides.

Statistical analysis was performed using the ProStaR software [26]. Proteins identified in the contaminant database, and proteins identified by MS/MS and quantified in less than three replicates of one condition were discarded. After log2 transformation, abundance values were normalized by condition using variance stabilizing normalization, before missing value imputation (slsa algorithm for partially observed values in the condition and DetQuantile algorithm for totally absent values in the condition). Statistical testing was then conducted using limma, whereby differentially expressed proteins were sorted out using a fold change cut-off of 5 and a *p*-value cut-off of 0.01, leading to a FDR inferior to 1% according to the Benjamini–Hochberg estimator.

The mass spectrometry proteomics data have been added to the ProteomeXchange Consortium via the PRIDE [27] partner repository with the following dataset identifier PXD045450 and 10.6019/PXD045450.

To determine biological processes in which RSK3 partners are involved mass spectrometry results were analyzed with the DAVID online tool (https://david.ncifcrf.gov/) [28].

### Proteasome analysis

Proteasome activity assay was performed in HMECT infected with pWZL or pWZL /RSK3 vector and selected for one week with neomycin. Cells were plated in 96-well plates at 5 × 10^3^ cells/well and analyzed with Cell-based Proteasome-Glo Chymotrypsin-like assay (Promega) according to the manufacturer’s instructions. The plates were read using a plate reader (Infinite M1000 Pro, Tecan). Proteasome enrichment assay was performed on 2 × 10^7^ HMECT using the Cyclex Proteasome enrichment and activity kit (MBL Life science CY-7002) and visualized by Western blot.

### Intraductal transplantation method

The intraductal xenograft model was carried out as previously described [29, 30]. Briefly, 10-week-old virgin female SCID mice were anesthetized (2% isofluorane, 2 L/min air), nipples of both inguinal glands #4 were snipped and 1.5 × 104 MCF10DCIS.com cells in 2 μl phosphate-buffered saline were injected in each gland. Mice were sacrificed between 4 and 5 weeks after injection by cervical dislocation. Immediately after being euthanized, mammary glands were excised and processed for further study. The animal facility was granted approval (C-75–12-01) given by the French Administration.

### Histological and immunofluorescence analysis of mouse tissue sections

Whole-mount carmine and hematoxylin and eosin (H&E) staining of tissue sections were performed as described in [31]. After whole mount staining, image acquisition was performed with an A1R Nikon confocal binocular microscope. To retrieve antigens on paraffin embedded tissue samples, sections were incubated for 20 min in 10 mM sodium citrate buffer, pH 6.0 at 90 °C. Then, after 60 min incubation in 5% fetal calf serum, sections were incubated overnight with diluted primary antibodies, washed and further incubated for 2 h at room temperature with appropriate secondary antibodies. Quantification was performed from 9 to 23 images of tumor sections from two to three different mammary glands.

### Statistical analysis

Statistical analysis was performed and graphs were created with Graph Pad Prism 9.4.1 and statistical test used are directly indicated in the figure legends. Data were normalized against the mean value of controls of each experiment.

## Results

### RSK3 inhibits human mammary epithelial cell senescence induced by TGFβ

**Fig. 1:**
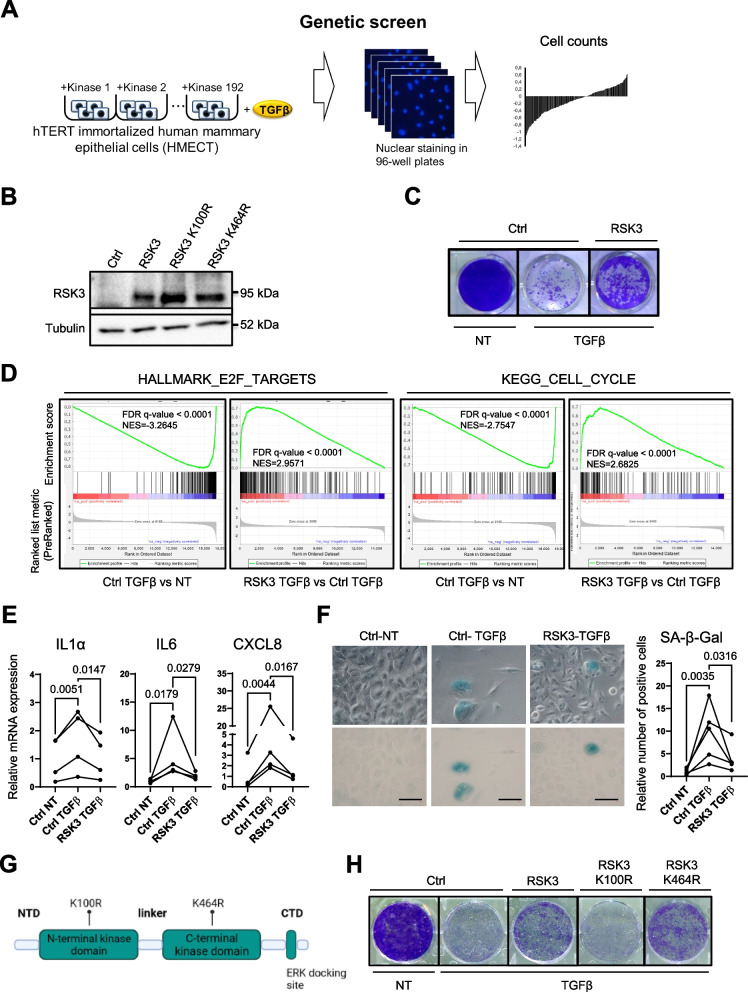
RSK3 inhibits TGFβ-induced senescence. **A** Schematic diagram of the genetic screen used. hTERT-immortalized human mammary epithelial cells (HMECT) were seeded in 96-well plates and infected with one kinase per well (*n* = 2 per kinase). After 24 h, cells were treated with TGFβ, and 72 h later cells were stained with Hoechst 33342 nucleic acid stain. Plates were read under a Cytell system and ranked according to the nuclei count. **B**-**F** HMECT were infected with pWZL/RSK3 (RSK3) or control vector pWZL (Ctrl), selected for 1 week with neomycin and plated for indicated assays, followed by TGFβ treatment (TGFβ) or not (NT). **B** Western blot validation of RSK3 overexpression. **C** Crystal violet assay for cell density 10 days after treatment with TGFβ (representative of 5 experiments). **D** GSEA enrichment plots related to cell proliferation. GSEA has been done on transcriptome data comparing global gene expression between TGFβ-treated HMECT-pWZL and non-treated HMECT-pWZL conditions (Ctrl TGFβ vs NT) or TGFβ-treated HMECT-pWZL/RSK3 and TGFβ-treated HMECT-pWZL conditions (RSK3 TGFβ vs Ctrl TGFβ). For each condition three independent experiments have been analyzed by transcriptomic (*n* = 3). False Discovery Rate (FDR) pValue and Normalized Enrichment Score (NES) are indicated. **E** RT-QPCR was performed 5 days after TGFβ treatment (*n* = 4 independent experiments). **F** SA-β-Gal assay, 7 days after treatment with TGFβ (*n* = 5 independent experiments). Scale bar = 50 μM. **G** Domains of RSK3 and mutated sites to generate kinase-deficient mutants are represented. Two kinase domain mutants were generated. **H** HMECT were infected with pWZL/RSK3 (RSK3), pWZL/RSK3 K100R (RSK3 K100R), pWZL/RSK3 K464R (RSK3 K464R) and pWZL control (Ctrl). Crystal violet assay for cell density 10 days after treatment with TGFβ (representative of 3 experiments). Ratio-paired t-test was used to determine statistical significance

In order to identify kinases that may impact TGFβ-induced senescence, we performed a genetic screen using a viral expression library of 192 active kinases [20] in hTERT-immortalized HMECs we have generated from several donors (HMECT). Kinases were overexpressed in separate wells containing HMECT cells, and the following day, cells were treated with TGFβ. The number of nuclei was counted two days after to obtain a list of kinases potentially inhibiting TGFβ-induced senescence (Fig. 1A and Supplemental Table 3). PIP5K1B, ACVR1, SGK, TK1, CHEK1, CSNK1G2, CDK4, PAPSS1, RPS6KA2, OXSR1 were identified as the top 10 hits. Finally, validation experiments confirmed that RPS6KA2 (Ribosomal Protein S6 Kinase) or RSK3 as a potential inhibitor of TGFβ-induced senescence. This kinase belongs to the RSK family of serine/threonine kinases that regulate diverse cellular processes, including cell survival and proliferation in cancer cells [32], albeit it has never been linked to senescence or TGFβ. This initial result was further confirmed after validation of the constitutive expression of the RSK3 protein (Fig. 1B), which largely rescued the decreased cell density induced by TGFβ (Fig. 1C). In addition, RSK3 partly prevented loss of EdU incorporation (Supplemental Figure 1A), loss of the Ki67 proliferation marker (Supplemental Figure 1B) and cyclin-dependent kinase inhibitor CDKN1A increase (Supplemental Figure 1C), induced by TGFβ treatment. Transcriptomic analyses and Gene Set Enrichment Analysis (GSEA) also demonstrated that the decreased molecular signatures linked to proliferation arrest (Kegg Cell Cycle and Hallmark E2F targets) induced by TGFβ were largely prevented upon RSK3 expression (Fig. 1D), also supporting that RSK3 inhibits TGFβ-induced proliferation arrest. RSK3 expression also decreased other marks of cellular senescence induced by TGFβ: SASP components expression (Fig. 1E) and senescence-associated-β-galactosidase activity (SA-β-Gal) (Fig. 1F).

As RSK3 has two functional kinase domains, a carboxyl-terminal domain and an amino-terminal domain [33], we mutated each of these kinase domains [34, 35] and expressed the corresponding RSK3 mutants in HMECT cells (Fig. 1B, G). In contrast to the expression of RSK3 or RSK3 C-terminal kinase domain mutant (K464R) that rescued TGFβ-induced senescence, the expression of the N-terminal kinase domain mutant (K100R) impaired this rescue (Fig. 1H). Together, these results support that RSK3 prevents TGFβ-induced senescence through the activity of its N-terminal kinase domain.

### Endogenous RSK3 decreases after TGFβ treatment in a SMAD3-dependent manner

**Fig. 2:**
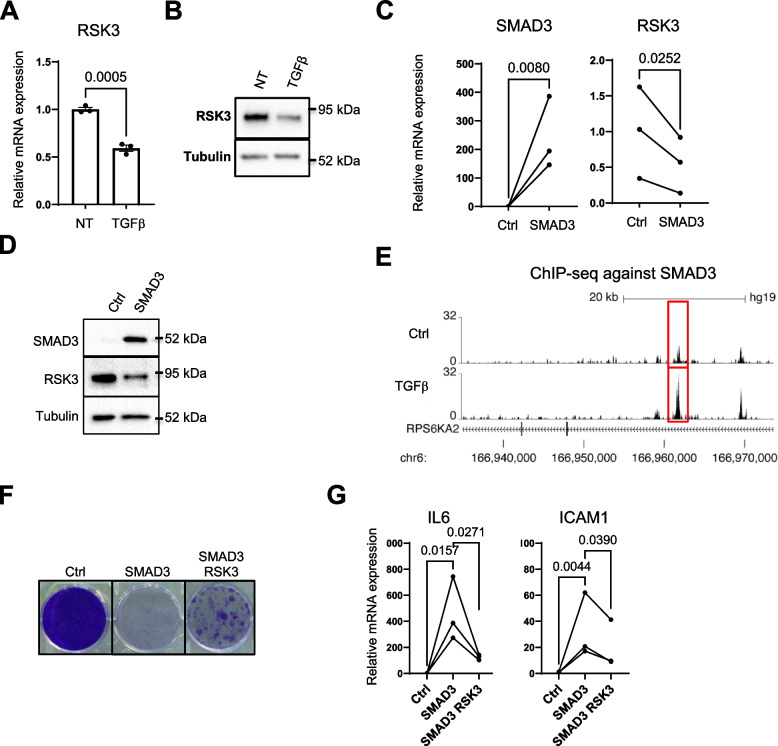
TGFβ represses RSK3 expression through SMAD3. **A** RSK3 expression decreased after TGFβ treatment. Transcriptome analyses (*n* = 3) were performed 48 h after cell treatment with TGFβ and RSK3 mRNA levels are shown. Statistical significance was determined with unpaired t-test. **B** Western blot validation of RSK3 protein decrease. **C**, **D** Cells were infected with pLPCX (Ctrl), or pLPCX/SMAD3 (SMAD3). **C** SMAD3 constitutive expression (left) and RSK3 gene expression (right). RT-QPCR was performed 72 h after infection (*n* = 3 independent experiments). **D** Western blot to assess RSK3 decrease induced by SMAD3 constitutive expression 3 days after infection (representative of 2 experiments). **E** ChIP-seq data analysis of SMAD3 binding in HMEC treated by TGFβ (TGFβ) or not (Ctrl) were visualized in UCSC genome browser after BigWig files uploading (GSM2807302 for non-treated cells and GSM2807304 for TGFβ-treated cells). **F**-**G** HMECT were infected with pWZL or pWZL/RSK3 and selected for 1 week with neomycin and next were infected with pLPCX or pLPCX/SMAD3. **F** Crystal violet assay for cell density 10 days after overexpression of SMAD3 (representative of 2 experiments). **G** Levels of mRNA encoding SASP factors. RT-QPCR was performed 3 days after infection with SMAD3 (*n* = 3 independent experiments). Ratio-paired t-test was used to determine *p*-value

Interestingly, and according to transcriptomic data, RSK3 mRNA levels decreased upon TGFβ stimulation (Fig. 2A), suggesting that endogenous RSK3 could be involved in TGFβ signaling. This decrease in mRNA was also observed at the protein level (Fig. 2B). As canonical TGFβ signaling is mediated by the SMAD (Small Mothers Against Decapentaplegic) transcription factors [36], we tested the impact of constitutively expressing SMADs (Fig. 2C, Supplemental Figure 2A) on RSK3 levels. The constitutive expression of SMAD3, but not that of SMAD2 or SMAD4, reduced RSK3 mRNA and protein levels (Fig. 2C-D, Supplemental Figure 2A). This effect of SMAD3 on RSK3 expression may be direct as it binds to the *RSK3* gene and this binding is further increased by TGFβ treatment as detected in ChIP-seq data [37] (Fig. 2E).

In addition, only the constitutive expression of SMAD3 induced proliferation arrest and strongly increased the expression of SASP factor IL6 (Fig. 2F-G, Supplemental Figure 2B-C). This premature senescence induced by SMAD3 was partly reverted by RSK3 overexpression as it largely prevented proliferation arrest (Fig. 2F) and SASP induction (Fig. 2G) by SMAD3. According to these results, RSK3 may be an actor of TGFβ signaling as its expression decreased during TGFβ treatment, in a SMAD3-dependent manner, and as its constitutive expression rescued cells from TGFβ- as well as SMAD3-induced premature cellular senescence.

### RSK3 reverts senescence by inhibiting NF-κB transcription factors

**Fig. 3:**
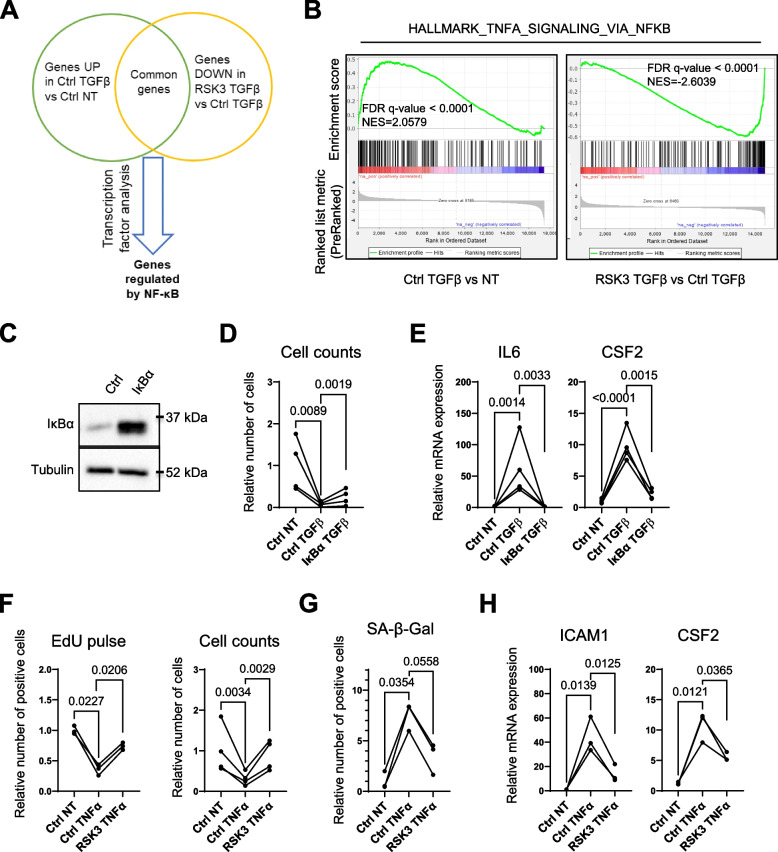
RSK3 reverts senescence by modulating NF-κB activity. **A**, **B** Transcriptome analyses (*n* = 3) were performed 48 h after cell treatment with TGFβ. **A** Strategy to identify transcriptional program altered by TGFβ and RSK3 expression. Identification by Venn diagram and TFacts software of NF-κB target genes found in common between up-regulated genes in TGFβ-treated HMECT-pWZL (Ctrl TGFβ) versus non-treated HMECT-pWZL (Ctrl NT) conditions and down-regulated genes in TGFβ-treated HMECT-pWZL/RSK3 (RSK3 TGFβ) versus TGFβ-treated HMECT-pWZL (Ctrl TGFβ) conditions. **B** GSEA enrichment plots related to NF-κB signaling. **C**, **D** HMECT were infected with pBABE (Ctrl) or pBABE/IκBα super repressor (IκBα) and selected for 3 days with puromycin, then plated for indicated assays, followed by TGFβ treatment on the next day when indicated. **C** Western blot validation of IκBα overexpression. **D** Cell counts (*n* = 4 independent experiments). **E** RT-QPCR of SASP factor expression (*n* = 4 independent experiments). (F–H) HMECT were infected with pWZL/RSK3 (RSK3) or control pWZL (Ctrl) vectors, selected for 1 week with neomycin and plated for indicated assays, followed by TNFα treatment (TNFα) or not (NT) on the next day. **F** EdU pulse and cell count. EdU was added 2 days after treatment with TNFα (*n* = 3 independent experiments). Cell counts were performed 7 days after TNFα treatment (*n* = 4 independent experiments). **G** SA-β-Gal assay was performed 7 days after treatment with TNFα (*n* = 3 independent experiments). **H** RT-QPCR was performed on the indicated SASP encoding mRNAs (*n* = 3 independent experiments). Ratio-paired t-test was used to determine statistical significance

To decipher the mechanism(s) by which RSK3 reverts induced senescence in HMECT we performed common transcription factor analysis using TFacts software [38] on genes whose expression changed upon TGFβ stimulation and was reverted by RSK3, according to our transcriptomic analysis. Members of the NF-κB (nuclear factor-kappa B) family of transcription factors were ranked first as potential transcriptional regulators of genes upregulated by TGFβ treatment when compared to non-treated cells, and downregulated in TGFβ-treated cells expressing RSK3 when compared to control TGFβ-treated cells (Fig. 3A and Supplemental Table 4). The NF-κB family of transcription factors is well known for promoting cellular senescence and the expression of the SASP [39–42]. In addition to the increased expression in some NF-κB-dependent SASP factors upon TGFβ stimulation and its reversion by RSK3 (Fig. 1E, Supplemental Figure 3), we detected a global positive regulation NF-κB signaling and its reversal by RSK3 using GSEA (Fig. 3B). To investigate whether NF-κB inhibition mimicked effects observed with RSK3, we constitutively expressed a stabilized form of IκBα (nuclear factor of kappa light polypeptide gene enhancer in B-cell inhibitor, alpha) (Fig. 3C), a protein that sequesters NF-κB in the cytoplasm [43]. Strikingly, inhibition of NF-κB by IκBα expression partly reverted not only SASP expression but also TGFβ-induced proliferation arrest (Fig. 3D-E). These results support that RSK3 could inhibit TGFβ-induced senescence by decreasing NF-κB activity.

We then decided to investigate the impact of RSK3 on a canonical and direct activator of the NF-κB pathway, namely TNFα (Tumor Necrosis Factor α) [44]. The constitutive expression of RSK3 blocked the proliferation arrest (Fig. 3F), SA-β-Gal activity (Fig. 3G) and SASP upregulation (Fig. 3H) induced by TNFα, supporting that RSK3 inhibits NF-κB activity. Overall, these results support that RSK3 prevents senescence by inhibiting NF-κB, a master regulator of cellular senescence and its SASP.

### RSK3 stabilizes IκBα by interacting with and repressing the proteasome

**Fig. 4:**
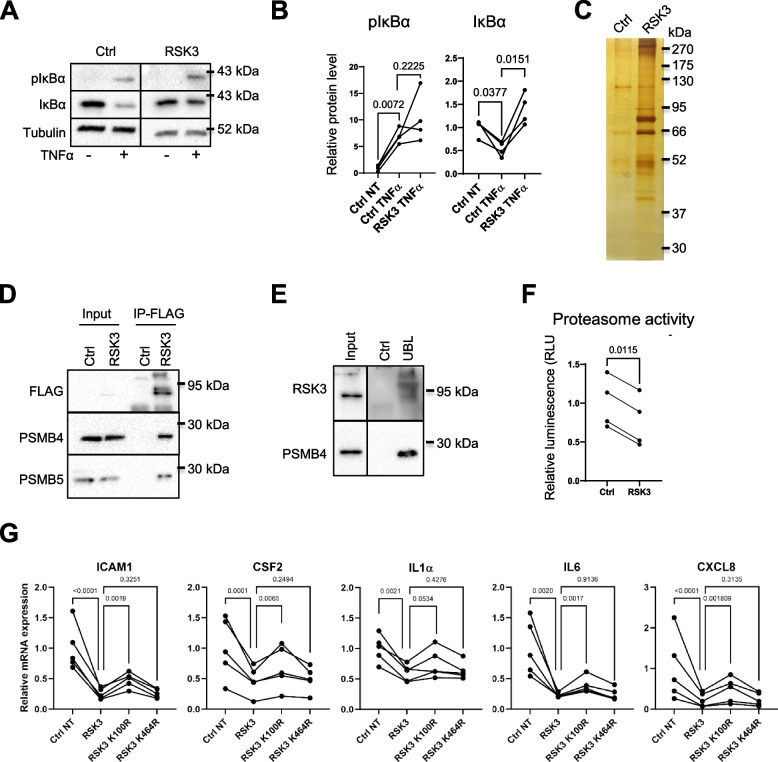
RSK3 controls proteasome activity and IκBα degradation. **A**, **B** HMECT were infected with pWZL/RSK3 (RSK3) or control pWZL vector (Ctrl), selected for 1 week with neomycin and plated for indicated assays. **A** Western blot characterization of pIκBα and IκBα levels. Cells were stimulated with TNFα for 5 min. **B** Quantification of the Western blots of panel A, normalization with tubulin (*n* = 4, ratio paired t-test was used to determine statistical significance). **C**, **D** 293GP cells were transfected with pFLAG-CMV2 (Ctrl) or pFLAG-CMV2/RSK3 (RSK3) and 48 h after collected for FLAG immunoprecipitation. **C** Silver staining of FLAG-immunoprecipitated proteins before mass spectromety analysis. **D** FLAG-immunoprecipitation and Western blot against the indicated tag and proteins (representative of 3 independent experiments). **E** Proteasome enrichment. Assay was performed in HMECT with Cyclex Proteasome enrichment kit and a further Western blot was performed (representative of 3 independent experiments) Ctrl = control resin, UBL = UBL resin, binding to the proteasome. **F** Proteasome activity assay performed in HMECT constitutively expressing or not RSK3 (*n* = 4 independent experiment, ratio-paired t-test was used to determine statistical significance). **G** RT-QPCR of NF-κB-dependent SASP encoding mRNA in HMECT expressing or not RSK3 and RSK3 mutants (*n* = 3 independent experiments). Ratio-paired t-test was used to determine statistical significance

A critical step in the activation of the NF-κB pathway is the degradation of IκBα [45]. We performed a Western blot to detect potential changes in phosphorylation and in the levels of IκBα, which depend on phosphorylation-induced proteasomal degradation upon TNFα stimulation [46, 47], following RSK3 expression. We observed decreased IκBα degradation, while its phosphorylation, which should lead to its degradation, was not altered significantly upon RSK3 constitutive expression (Fig. 4A-B).

To better understand how RSK3 might interfere with IκBα degradation, we conducted immunoprecipitation experiments to purify the RSK3-Flag protein and its partners (Fig. 4C). Mass spectrometry-based proteomics was then performed to identify potential partners. Importantly, some known RSK3 partners were detected, such as ERK1 and ERK2 [48], thus validating the relevance of our results. Pathway analysis of RSK3-interacting proteins revealed proteasome components involved in IκBα degradation among the top enriched processes (Supplemental Figure 4A, Supplemental Tables 5 and 6). Interactions between RSK3 and PSMB4 and PSMB5, core proteasome components, were confirmed (Fig. 4D). In addition, inhibition of IκBα degradation by RSK3 expression upon TNFα treatment was mimicked by MG132 proteasome inhibitor (Supplemental Figure 4B).

Inversely, endogenous RSK3 was found in the proteasome enriched fraction of HMECT (Fig. 4E) and proteasome activity was decreased in HMECT by about 25% in cells overexpressing RSK3 (Fig. 4F). Accordingly, numerous classical NF-κB targets were repressed in RSK3-overexpressing HMECT without any stimulation (Fig. 4G). As for TGFβ-induced senescence bypass (Fig. 1H), the RSK3 N-terminal kinase domain mutant (K100R) displayed an impaired ability to inhibit the expression of NF-κB targets (Fig. 4G), in contrast to the C-terminal kinase domain mutant (K464R) that behaved as WT RSK3 (Fig. 4G).

Hence, these results support that RSK3 interacts with and decreases the activity of the proteasome, resulting in IκBα stabilization and in a decrease in NF-κB activity.

### RSK3 sustains EMT during senescence escape and it correlates with TGFβ signaling and EMT in human breast tumors

**Fig. 5:**
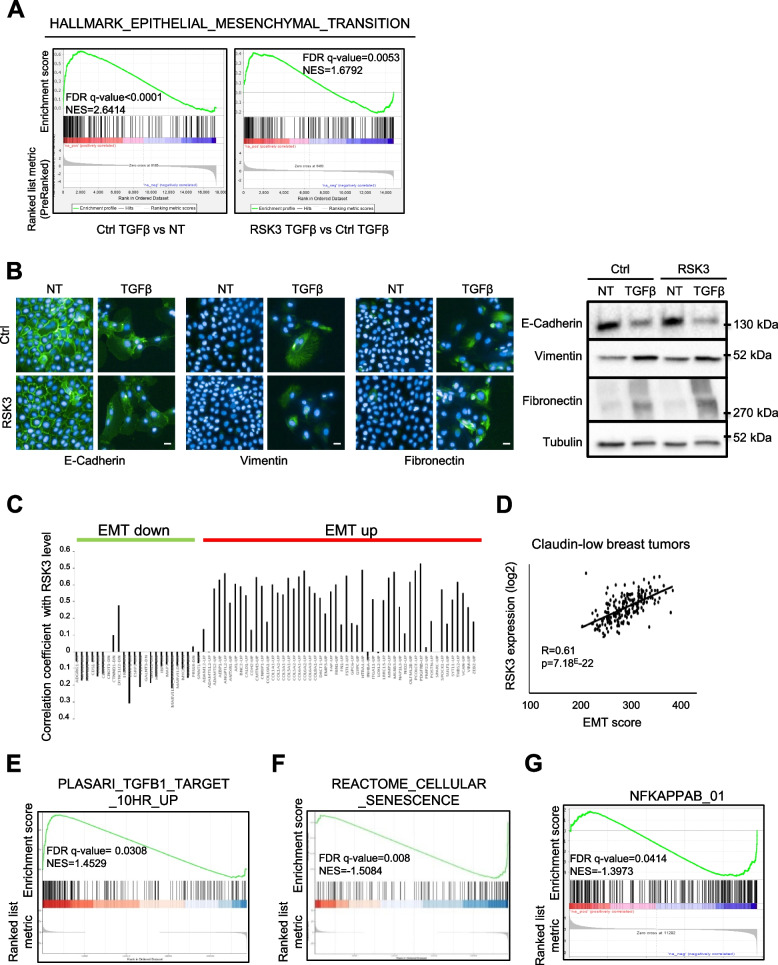
RSK3 sustains EMT during senescence escape and it correlates with TGFβ signaling and EMT in human breast tumors. **A** GSEA was performed on the data comparing TGFβ-treated HMECT-pWZL to non-treated HMECT-pWZL conditions (Ctrl TGFβ vs NT) and TGFβ-treated HMECT-pWZL/RSK3 to TGFβ-treated HMECT-pWZL conditions (RSK3 TGFβ vs Ctrl TGFβ). False Discovery Rate (FDR), pValue and Normalized Enrichment Score (NES) are indicated. **B** Immunofluorescence staining was performed 5 days after treatment with TGFβ (representative of 2 experiments, scale bar = 20 μm) (left). Western Blot exibiting changes in EMT markers (right) (Representative of 3 experiments). **C**-**G** Analysis of the METABRIC breast cancer gene expression database. **C** Graph showing Pearson’s correlation coefficients between RSK3 expression and the expression of genes known to be down- or up-regulated during EMT in breast tumors. **D** Correlation between EMT score and RSK3 expression in Claudin-low breast tumors. **E**–**G** Claudin-low breast tumors were divided into 2 groups by the median of RSK3 expression, the fold change between RSK3 high vs low was calculated for all the genes and GSEA was performed. GSEA enrichment plot related to TGFβ (**E**), cellular senescence (**F**) and NF-κB regulated genes (**G**) are shown

TGFβ was reported to induce not only senescence in HMECT, but also EMT as previously published [13]. According to our transcriptomic analysis we also observed an increase in the EMT molecular signature (Hallmark Epithelial Mesenchymal Transition) by GSEA (Fig. 5A), a decrease and altered localization of the E-cadherin epithelial marker, as well as an increase in vimentin and fibronectin mesenchymal markers (Fig. 5B). We then investigated whether RSK3 was also able to inhibit TGFβ-induced EMT. Its constitutive expression did not prevent TGFβ-induced EMT according to GSEA results (Fig. 5A) and did not lead to a decrease in levels of epithelial and mesenchymal markers (Fig. 5B). Hence, whereas RSK3 impedes TGFβ-senescence, the TGFβ-induced mesenchymal phenotype is sustained upon RSK3 expression.

Next, we explored RSK3 expression in a large cohort of human breast tumors and examined whether its expression profile was correlated with some molecular features, in particular EMT status and cellular senescence. For this purpose, we used the Molecular Taxonomy of Breast Cancer International Consortium (METABRIC) dataset consisting of 1904 breast tumors and their associated molecular profiles [49]. RSK3 expression was positively correlated with a majority of genes upregulated in EMT and negatively correlated with downregulated ones (Fig. 5C and Supplemental Table 7) [50]. For further analyses we focused on the Claudin-low subtype of breast cancer, characterized by a high EMT score [51] and we observed a significant correlation between RSK3 expression and the EMT score (Fig. 5D). Interestingly, Claudin-low breast tumors expressing high levels of RSK3 displayed increased TGFβ signaling (Fig. 5E), decreased cellular senescence (Fig. 5F) and decreased NF-κB (Fig. 5G) molecular signatures. RSK3 expression was also lower in TP53 mutated (Mut) than wild type (WT) Claudin-low breast tumors (Supplemental Figure 5), suggesting that in the case of Mut TP53, RSK3 alterations would be a redundant advantage.

These results support that RSK3 is expressed at higher levels in breast cancer cells displaying EMT marks where it could confer some growth advantage.

### Higher RSK3 expression increases invasion in an experimental mouse breast cancer model and correlates with invasiveness in human Claudin-low breast cancers

**Fig. 6:**
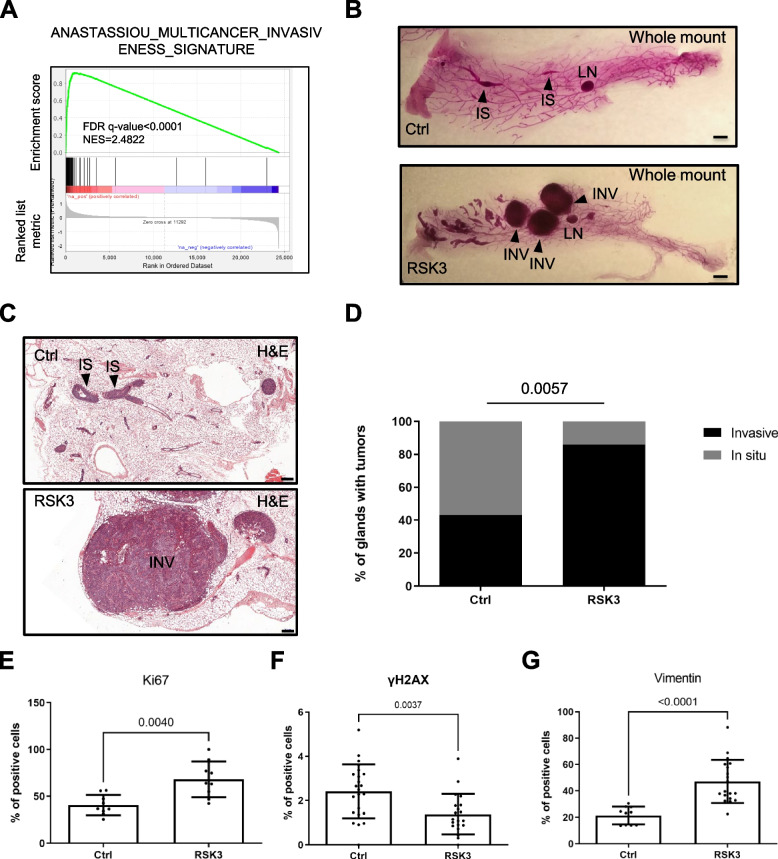
Higher RSK3 expression correlates with invasiveness in human Claudin-low breast cancers and increases invasion in an experimental mouse breast cancer model. **A** Analysis of the METABRIC breast cancer gene expression database. Claudin-low breast tumors were divided into 2 groups by the median of RSK3 expression, the fold change between RSK3 high vs low was calculated for all the genes and GSEA was performed. GSEA enrichment plot related to multicancer invasiveness. **B**-**G** Mammary glands were analyzed 4–5 weeks after injection of the indicated MCF10DCIS.com cell populations (Ctrl or constitutively expressing RSK3). **B** Whole-mount carmine-stained glands analyzed 4–5 weeks after injection of the indicated MCF10DCIS.com cell populations (Ctrl or constitutively expressing RSK3). IS, in situ; INV, invasive; LN, lymph node. Scale bar, 1.5 mm. **C** H&E staining of nipple-injected glands 4–5 weeks after injection of the indicated MCF10DCIS.com cell populations. Scale bar, 200 µm. **D** Phenotypic analysis of intraductal xenograft tumors of MCF10DCIS.com cells control or overexpressing RSK3. Analysis was based on whole-mount and H&E staining at 4–5 weeks after intraductal injection (Chi-square test). **E**–**G** Immunofluorescence against the indicated proteins were performed. Percentage of positive-cells were calculated after staining of the nucleus. **E** Ctrl *n* = 9, RSK3 = 9. **F** Ctrl *n* = 23, RSK3 *n* = 23. **G** Ctrl *n* = 10, RSK3 *n* = 19. Mann–Whitney test was used to determine statistical significance

The EMT process is generally involved in cell invasion [52]. To determine whether RSK3 expression, associated with EMT, is also be associated with cell invasion, we examined whether RSK3 expression was associated with molecular markers of invasion in human claudin-low breast tumors. We then divided Claudin-low breast tumors into 2 groups based on RSK3 expression levels and performed GSEA. High RSK3 expression was positively correlated with invasion in cancer (Fig. 6A).

Functional ability of RSK3 to promote breast cancer invasion was investigated using a xenograft mouse model based on the intraductal injection of low tumorigenic human breast cancer MCF10DCIS.com cells. This model spontaneously recapitulates the transition from in situ to invasive stages of breast cancer progression [29–31]. Strikingly, RSK3 constitutive expression increased the proportion of invasive breast tumors (Fig. 6B-D). Furthermore, tumors with RSK3 overexpression displayed more proliferating cells according to Ki67 staining (Fig. 6E, Supplemental Figure 6) and less DNA damaged cells according to γH2AX staining (Fig. 6F, Supplemental Figure 7) than control tumors, suggesting that RSK3 decrease senescent features in RSK3-overexpressing tumors. In addition, RSK3 tumors displayed increased EMT-positive cells according to vimentin staining (Fig. 6G, Supplemental Figure 8).

Collectively, these data suggest that the higher expression of RSK3 found in EMT high, Claudin-low breast cancer accelerates breast tumor progression.

## Discussion

Although critical pathways regulating cellular senescence are well characterized, mechanisms that can both inhibit senescence and promote the malignant conversion of human epithelial cells are poorly understood. TGFβ can induce senescence and its anti-tumoral effects or tumor formation and progression. In order to identify a potential pro-tumoral switch, we screened a library of active kinases in the context of TGFβ-induced senescence and EMT. We identified an understudied kinase, namely RSK3, able to inhibit TGFβ-induced senescence. RSK3 seems to be part of TGFβ signaling as its expression decreased following TGFβ stimulation. Mechanistically the downregulation of RSK3 by TGFβ was mediated by the SMAD3 transcription factor. In line with our results, SMAD3 was reported to mediate senescence [53, 54]. Loss of SMAD3 and decreased senescence were then shown to promote skin tumor progression [53].

To discover the mechanism(s) by which RSK3 regulates senescence, we combined transcriptomic and affinity purification coupled to mass spectrometry-based proteomics to identify senescence pathway(s) impacted by RSK3, and RSK3 partners that could impact those senescence pathway(s). These two approaches concomitantly led to the identification of the NF-κB pathway. NF-κB transcription factors are well-known key regulators of cellular senescence, notably by regulating the SASP [39–42, 55]. We then conducted several experiments to confirm the role of the NF-κB pathway in regulating TGFβ-induced senescence and its reversal by RSK3. Phosphorylation-induced degradation of IκBα is considered to be the main step to activate NF-κB factors and most of the known regulators of the NF-κB pathway directly or indirectly impact IκBα phosphorylation [45]. Although a recent study suggested that RSK3 could interact with IκBα and promote its phosphorylation [56] we did not observe any promotion of IκBα phosphorylation by RSK3, nor did we identify IκBα among RSK3 interactants. Instead, we observed that stability of IκBα increased, though its phosphorylation was unaffected, suggesting that RSK3 impacts the degradation of IκBα instead of its phosphorylation. Supporting this hypothesis, several proteasome components, the machinery involved in IκBα degradation [45], were identified as partners of RSK3. In addition, endogenous RSK3 was affinity-precipitated with the proteasome and its constitutive expression decreased proteasome activity and decreased NF-κB target gene expression, supporting that RSK3 inhibits NF-κB activity by decreasing proteasome activity. Senescence reversal by inhibition of NF-κB pathway by decreasing proteasome activity seems to be dependent on action of the N-terminal domain of this kinase. It is largely believed that the C-terminal domain is involved in autophosphorylation and activation of RSK; however, the N-terminal domain is responsible for the phosphorylation of exogenous substrates [48].

TGFβ can induce EMT of normal human epithelial cells and this effect can also occur in the context of senescence induction in these cells, according to our results, by creating a unique hybrid EMT/senescence state with both potentially pro- and anti- tumoral capacities. Strikingly, RSK3 prevented senescence while sustaining EMT, suggesting that it not only prevents proliferation arrest but it could also favor a malignant switch by tipping the balance. This is also supported by the profile of expression of RSK3 in human breast tumors, which was strongly correlated with TGFβ signaling, EMT signature and invasion, and inversely correlated with cellular senescence and NF-κB signature in a large set of Claudin-low breast tumors. Moreover, RSK3 accelerated the breast cancer in situ – invasive switch in mice with decreased senescent marks and increased EMT features further supporting a malignant twist promoted by this kinase.

Aside from a few articles supporting that RSK3 regulates cell death, mostly by protecting cancer cells from cell death, for instance by promoting resistance to (i) PI3K inhibitors [57] or cisplatin [58] in breast cancer, (ii) EGFR inhibitors in the context of pancreatic cancer [59] or (iii) BET inhibitors in lung cancer [60] and occasionally promoting cell death [61], very little is known on this kinase. Our results highlight its ability to overcome cellular senescence, to maintain an EMT phenotype and to promote tumor invasion. Thus our results and the above-described literature [57–61] emphasize the interest of further investigating the effects of RSK3 inhibition in different type of cancers, alone, at least in claudin-low breast tumors, or in combination with drugs targeting pro-tumoral pathways, for many types of tumors. To do so, there is an urgent need to develop specific RSK3 inhibitors in contrast the currently known pan-RSK inhibitors [62, 63]. Our results also highlight a never reported hybrid EMT/senescence state, however, whether this state exists only in the context of TGFβ signals or whether it can also occur in other pro-senescence or pro-EMT contexts remains to be investigated.

## Conclusions

In conclusion, we have identified a tumor promoting role for the RSK3 kinase in breast cancer. Our findings indicate that RSK3 is part of the TGFβ pathway, as its expression is regulated by SMAD3. RSK3 is able to overcome the anti-tumoral effect of TGFβ-induced senescence, without impacting pro-tumoral EMT. Mechanistically RSK3 supresses senescence by downregulating the senescence master regulator NF-κB pathway, by supressing proteosomal degradation of its inhibitor IκBα. These results pave the way for future studies centered on RSK3 and on the development of biomarkers or chemical inhibitors for more precise and effective claudin-low breast cancer treatments.

### Supplementary Information

**Additional file 1.**

## Data Availability

Transcriptomic datasets are available in GEO GSE243320. The mass spectrometry proteomics data have been deposited to the ProteomeXchange Consortium with the dataset identifier PXD045450 and 10.6019/PXD045450. The source data underlying the different figures are available upon request to the corresponding author.

## References

[1] Bartkova J, Rezaei N, Liontos M, Karakaidos P, Kletsas D, Issaeva N (2006). Oncogene-induced senescence is part of the tumorigenesis barrier imposed by DNA damage checkpoints. Nature.

[2] Prieur A, Peeper DS (2008). Cellular senescence in vivo: a barrier to tumorigenesis. Curr Opin Cell Biol.

[3] Coppé J-P, Patil CK, Rodier F, Sun Y, Muñoz DP, Goldstein J (2008). Senescence-associated secretory phenotypes reveal cell-nonautonomous functions of oncogenic RAS and the p53 tumor suppressor. PLoS Biol.

[4] Gorgoulis V, Adams PD, Alimonti A, Bennett DC, Bischof O, Bishop C (2019). Cellular senescence: defining a path forward. Cell.

[5] Birch J, Gil J (2020). Senescence and the SASP: many therapeutic avenues. Genes Dev.

[6] Schmitt CA, Wang B, Demaria M (2022). Senescence and cancer — role and therapeutic opportunities. Nat Rev Clin Oncol.

[7] Cipriano R, Kan CE, Graham J, Danielpour D, Stampfer M, Jackson MW (2011). TGF-beta signaling engages an ATM-CHK2-p53-independent RAS-induced senescence and prevents malignant transformation in human mammary epithelial cells. Proc Natl Acad Sci U S A.

[8] Canino C, Mori F, Cambria A, Diamantini A, Germoni S, Alessandrini G (2012). SASP mediates chemoresistance and tumor-initiating-activity of mesothelioma cells. Oncogene.

[9] Yoshimoto S, Loo TM, Atarashi K, Kanda H, Sato S, Oyadomari S (2013). Obesity-induced gut microbial metabolite promotes liver cancer through senescence secretome. Nature.

[10] Azazmeh N, Assouline B, Winter E, Ruppo S, Nevo Y, Maly A (2020). Chronic expression of p16(INK4a) in the epidermis induces Wnt-mediated hyperplasia and promotes tumor initiation. Nat Commun.

[11] Hosobuchi M, Stampfer MR (1989). Effects of transforming growth factor β on growth of human mammary epithelial cells in culture. Vitr Cell Dev Biol.

[12] Miettinen PJ, Ebner R, Lopez AR, Derynck R (1994). TGF-β induced transdifferentiation of mammary epithelial cells to mesenchymal cells: involvement of type I receptors. J Cell Biol.

[13] Lindley LE, Briegel KJ (2010). Molecular characterization of TGFbeta-induced epithelial-mesenchymal transition in normal finite lifespan human mammary epithelial cells. Biochem Biophys Res Commun.

[14] Chaffer CL, San Juan BP, Lim E, Weinberg RA (2016). EMT, cell plasticity and metastasis. Cancer Metastasis Rev.

[15] Hoare M, Ito Y, Kang T-W, Weekes MP, Matheson NJ, Patten DA (2016). NOTCH1 mediates a switch between two distinct secretomes during senescence. Nat Cell Biol.

[16] Hao Y, Baker D, Ten Dijke P (2019). TGF-β-mediated epithelial-mesenchymal transition and cancer metastasis. Int J Mol Sci.

[17] Zhang Y, Alexander PB, Wang X-F (2017). TGF-β family signaling in the control of cell proliferation and survival. Cold Spring Harb Perspect Biol.

[18] Fleuren EDG, Zhang L, Wu J, Daly RJ (2016). The kinome “at large” in cancer. Nat Rev Cancer.

[19] Counter CM, Hahn WC, Wei W, Caddle SD, Beijersbergen RL, Lansdorp PM (1998). Dissociation among in vitro telomerase activity, telomere maintenance, and cellular immortalization. Proc Natl Acad Sci U S A.

[20] Boehm JS, Zhao JJ, Yao J, Kim SY, Firestein R, Dunn IF (2007). Integrative genomic approaches identify IKBKE as a breast cancer oncogene. Cell.

[21] Katayama K, Fujiwara C, Noguchi K, Sugimoto Y (2016). RSK1 protects P-glycoprotein/ABCB1 against ubiquitin–proteasomal degradation by downregulating the ubiquitin-conjugating enzyme E2 R1. Sci Rep.

[22] Choy L, Skillington J, Derynck R (2000). Roles of autocrine TGF-beta receptor and Smad signaling in adipocyte differentiation. J Cell Biol.

[23] Papageorgis P, Cheng K, Ozturk S, Gong Y, Lambert AW, Abdolmaleky HM (2011). Smad4 inactivation promotes malignancy and drug resistance of colon cancer. Cancer Res.

[24] Casabona MG, Vandenbrouck Y, Attree I, Couté Y (2013). Proteomic characterization of Pseudomonas aeruginosa PAO1 inner membrane. Proteomics.

[25] Bouyssié D, Hesse A-M, Mouton-Barbosa E, Rompais M, Macron C, Carapito C (2020). Proline: an efficient and user-friendly software suite for large-scale proteomics. Bioinformatics.

[26] Couté Y, Bruley C, Burger T (2020). Beyond target-decoy competition: stable validation of peptide and protein identifications in mass spectrometry-based discovery proteomics. Anal Chem.

[27] Perez-Riverol Y, Bai J, Bandla C, García-Seisdedos D, Hewapathirana S, Kamatchinathan S (2022). The PRIDE database resources in 2022: a hub for mass spectrometry-based proteomics evidences. Nucleic Acids Res.

[28] Sherman BT, Hao M, Qiu J, Jiao X, Baseler MW, Lane HC (2022). DAVID: a web server for functional enrichment analysis and functional annotation of gene lists (2021 update). Nucleic Acids Res.

[29] Behbod F, Kittrell FS, LaMarca H, Edwards D, Kerbawy S, Heestand JC (2009). An intraductal human-in-mouse transplantation model mimics the subtypes of ductal carcinoma in situ. Breast Cancer Res.

[30] Nader GP de F, Agüera-Gonzalez S, Routet F, Gratia M, Maurin M, Cancila V, et al. Compromised nuclear envelope integrity drives TREX1-dependent DNA damage and tumor cell invasion. Cell. 2021;184:5230–5246.e22. 10.1016/j.cell.2021.08.035 34551315

[31] Lodillinsky C, Infante E, Guichard A, Chaligné R, Fuhrmann L, Cyrta J (2016). p63/MT1-MMP axis is required for in situ to invasive transition in basal-like breast cancer. Oncogene.

[32] Houles T, Roux PP (2018). Defining the role of the RSK isoforms in cancer. Semin Cancer Biol.

[33] Lara R, Seckl MJ, Pardo OE (2013). The p90 RSK family members: common functions and isoform specificity. Cancer Res.

[34] Shimamura A, Ballif BA, Richards SA, Blenis J (2000). Rsk1 mediates a MEK-MAP kinase cell survival signal. Curr Biol.

[35] Arthur JSC, Fong AL, Dwyer JM, Davare M, Reese E, Obrietan K (2004). Mitogen- and stress-activated protein kinase 1 mediates cAMP response element-binding protein phosphorylation and activation by neurotrophins. J Neurosci.

[36] Tzavlaki K, Moustakas A (2020). TGF-β Signaling. Biomolecules.

[37] Vervoort SJ, Lourenço AR, Tufegdzic Vidakovic A, Mocholi E, Sandoval JL, Rueda OM (2018). SOX4 can redirect TGF-β-mediated SMAD3-transcriptional output in a context-dependent manner to promote tumorigenesis. Nucleic Acids Res.

[38] Essaghir A, Toffalini F, Knoops L, Kallin A, van Helden J, Demoulin JB (2010). Transcription factor regulation can be accurately predicted from the presence of target gene signatures in microarray gene expression data. Nucleic Acids Res.

[39] Bernard D, Gosselin K, Monte D, Vercamer C, Bouali F, Pourtier A (2004). Involvement of Rel/Nuclear factor-κB transcription factors in keratinocyte senescence. Cancer Res.

[40] Ferrand M, Kirsh O, Griveau A, Vindrieux D, Martin N, Defossez PA (2015). Screening of a kinase library reveals novel pro-senescence kinases and their common NF-κB-dependent transcriptional program. Aging (Albany NY).

[41] Acosta JC, O’Loghlen A, Banito A, Guijarro MV, Augert A, Raguz S (2008). Chemokine signaling via the CXCR2 receptor reinforces senescence. Cell.

[42] Lopes-Paciencia S, Saint-Germain E, Rowell M-C, Ruiz AF, Kalegari P, Ferbeyre G (2019). The senescence-associated secretory phenotype and its regulation. Cytokine.

[43] Beg AA, Ruben SM, Scheinman RI, Haskill S, Rosen CA, Baldwin AS (1992). I kappa B interacts with the nuclear localization sequences of the subunits of NF-kappa B: a mechanism for cytoplasmic retention. Genes Dev.

[44] Liu T, Zhang L, Joo D, Sun S-C (2017). NF-κB signaling in inflammation. Signal Transduct Target Ther.

[45] Karin M, Ben-Neriah Y (2000). Phosphorylation meets ubiquitination: the control of NF-[kappa]B activity. Annu Rev Immunol.

[46] Alkalay I, Yaron A, Hatzubai A, Orian A, Ciechanover A, Ben-Neriah Y (1995). Stimulation-dependent IκBα phosphorylation marks the NF-κb inhibitor for degradation via the ubiquitin-proteasome pathway. Proc Natl Acad Sci U S A.

[47] Brown K, Gerstberger S, Carlson L, Franzoso G, Siebenlist U (1995). Control of I kappa B-alpha proteolysis by site-specific, signal-induced phosphorylation. Science.

[48] Roux PP, Richards SA, Blenis J (2003). Phosphorylation of p90 Ribosomal S6 Kinase (RSK) Regulates Extracellular Signal-Regulated Kinase Docking and RSK Activity. Mol Cell Biol.

[49] Curtis C, Shah SP, Chin SF, Turashvili G, Rueda OM, Dunning MJ (2012). The genomic and transcriptomic architecture of 2,000 breast tumours reveals novel subgroups. Nature.

[50] Mak MP, Tong P, Diao L, Cardnell RJ, Gibbons DL, William WN (2016). A patient-derived, pan-cancer EMT signature identifies global molecular alterations and immune target enrichment following epithelial-to-mesenchymal transition. Clin Cancer Res.

[51] Prat A, Parker JS, Karginova O, Fan C, Livasy C, Herschkowitz JI (2010). Phenotypic and molecular characterization of the claudin-low intrinsic subtype of breast cancer. Breast Cancer Res.

[52] Pastushenko I, Blanpain C (2019). EMT Transition States during Tumor Progression and Metastasis. Trends Cell Biol.

[53] Vijayachandra K, Lee J, Glick AB (2003). Smad3 regulates senescence and malignant conversion in a mouse multistage skin carcinogenesis model. Cancer Res.

[54] Bryson BL, Junk DJ, Cipriano R, Jackson MW (2017). STAT3-mediated SMAD3 activation underlies Oncostatin M-induced Senescence. Cell Cycle.

[55] Zdanov S, Bernard D, Debacq-Chainiaux F, Martien S, Gosselin K, Vercamer C (2007). Normal or stress-induced fibroblast senescence involves COX-2 activity. Exp Cell Res.

[56] Yoon HS, Choi SH, Park JH, Min JY, Hyon JY, Yang Y (2021). A Novel protein-protein interaction between RSK3 and IκBα and a new binding inhibitor that suppresses breast cancer tumorigenesis. Cancers (Basel).

[57] Serra V, Eichhorn PJA, García-García C, Ibrahim YH, Prudkin L, Sánchez G (2013). RSK3/4 mediate resistance to PI3K pathway inhibitors in breast cancer. J Clin Invest.

[58] Guan X, Meng X, Zhu K, Kai J, Liu Y, Ma Q (2022). MYSM1 induces apoptosis and sensitizes TNBC cells to cisplatin via RSK3–phospho-BAD pathway. Cell Death Discov.

[59] Milosevic N, Kühnemuth B, Mühlberg L, Ripka S, Griesmann H, Lölkes C (2013). Synthetic lethality screen identifies RPS6KA2 as modifier of epidermal growth factor receptor activity in pancreatic cancer. Neoplasia (United States).

[60] Kumari A, Gesumaria L, Liu YJ, Hughitt VK, Zhang X, Ceribelli M, et al. mTOR inhibition overcomes RSK3-mediated resistance to BET inhibitors in small cell lung cancer. JCI insight. 2023;8:e156657. 10.1172/jci.insight.156657 PMC10077471 36883564

[61] Wang M, Wan H, Wang S, Liao L, Huang Y, Guo L (2020). RSK3 mediates necroptosis by regulating phosphorylation of RIP3 in rat retinal ganglion cells. J Anat.

[62] Sun Y, Tang L, Wu C, Wang J, Wang C (2023). RSK inhibitors as potential anticancer agents: discovery, optimization, and challenges. Eur J Med Chem.

[63] Romeo Y, Roux PP (2011). Paving the way for targeting RSK in cancer. Expert Opin Ther Targets.

