# Supplementary material for: RSK3 switches cell fate: from stress-induced senescence to malignant progression
Source: J Exp Clin Cancer Res. 2023 Nov 27;42:318. doi: 10.1186/s13046-023-02909-5 (PMC10680185; doi:10.1186/s13046-023-02909-5)
Supplement: Supplementary file 1 — Additional file 1. [file 13046_2023_2909_MOESM1_ESM.pdf]

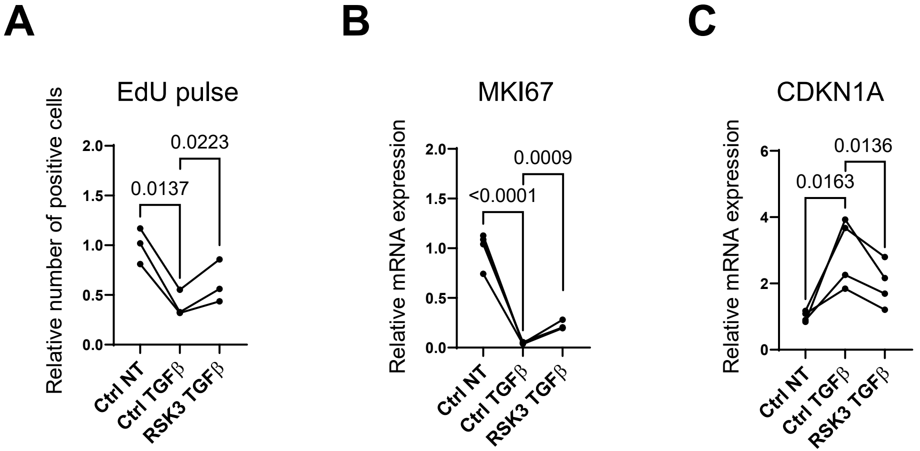

Figure S1. RSK3 inhibits TGFβ-induced proliferation arrest. HMECT were infected with pWZL/RSK3 (RSK3) or control vector pWZL (Ctrl), selected for 1 week with neomycin and plated for indicated assays, followed by TGFβ treatment at 0.5 ng/mL on the next day. (A) EdU pulse. Cells were plated at 1.5×10^3 cells/well in 96-well plates, 2 days after treatment with TGFβ EdU was added for 2 h before EdU staining (n = 3 independent experiments). (B to C) RT-QPCR was performed 48 h after TGFβ treatment (n = 4 independent experiments). Ratio paired t-test was used to determine statistical significance.

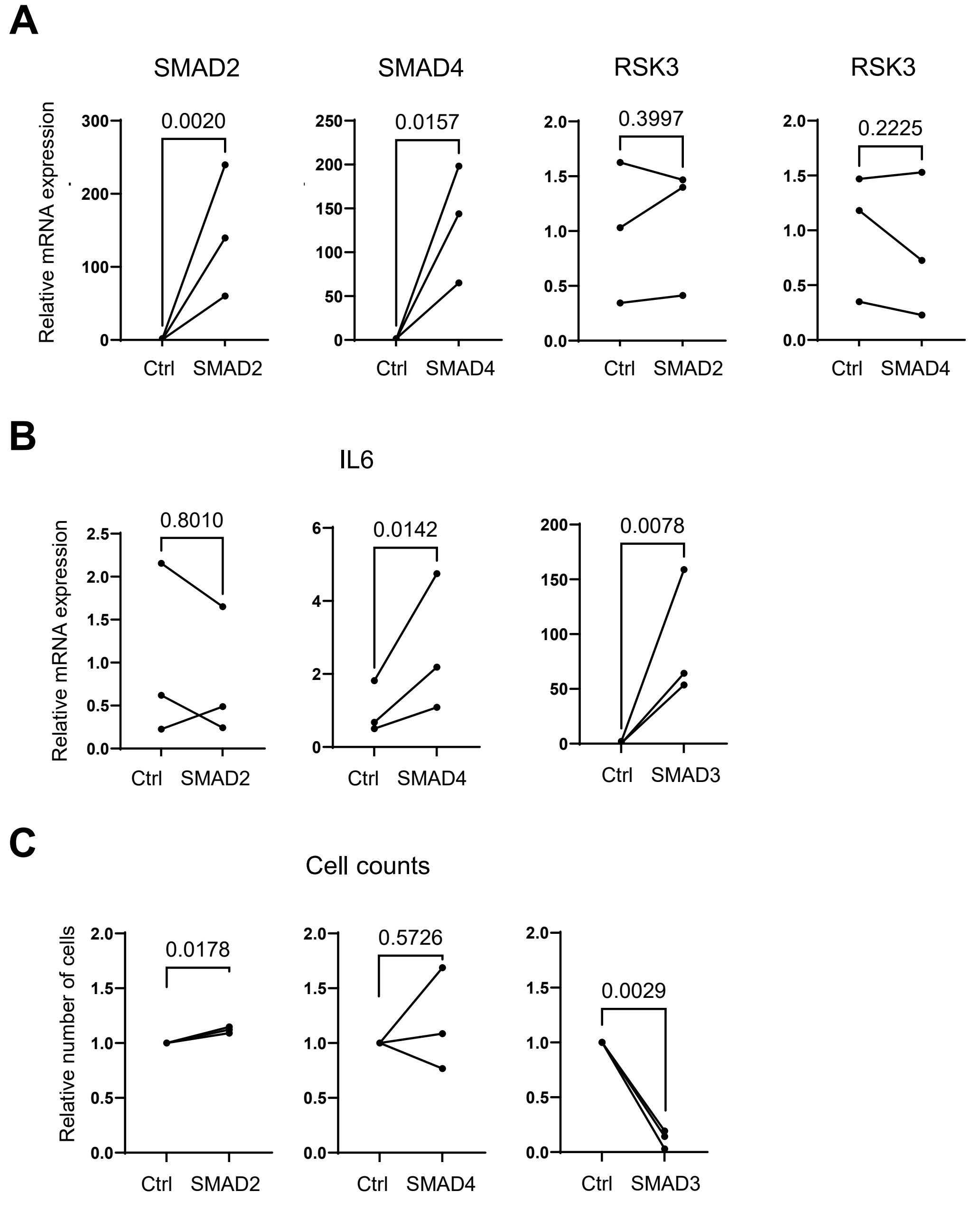

Figure S2. Cells were infected with pLPCX (Ctrl), pLPCX/SMAD2 (SMAD2), pLPCX/SMAD3 (SMAD3), pBABE (Ctrl) or pBABE/SMAD4 (SMAD4). (A) Validation of SMADs constitutive expression (left) and RSK3 expression (right) by RT-QPCR were performed 72 h after infection (n = 3 independent experiments). (B) IL6 mRNA levels were determined by RT-QPCR performed 72 h after infection (n = 3 independent experiments). (C) Impact of SMADs constitutive expression on cell number (n = 3 independent experiments). Ratio-paired t-test was used to determine p-value.

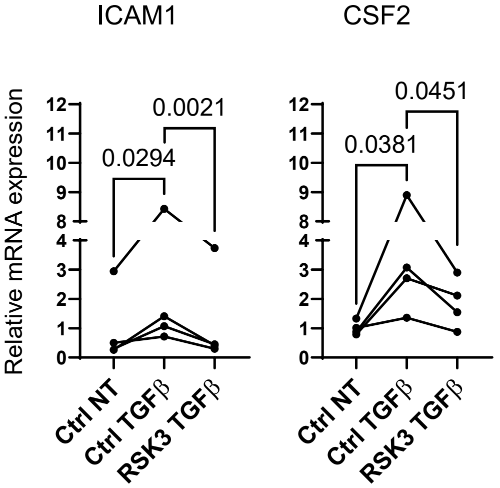

Figure S3. HMECT were infected with pWZL (Ctrl) or pWZL/RSK3 (RSK3) and selected for 1 week with neomycin, then plated for experiments, followed or not by TGFβ treatment on the next day. RT-QPCR against SASP-encoding mRNA, 48 h after the treatment (n = 4 independent experiments).

A

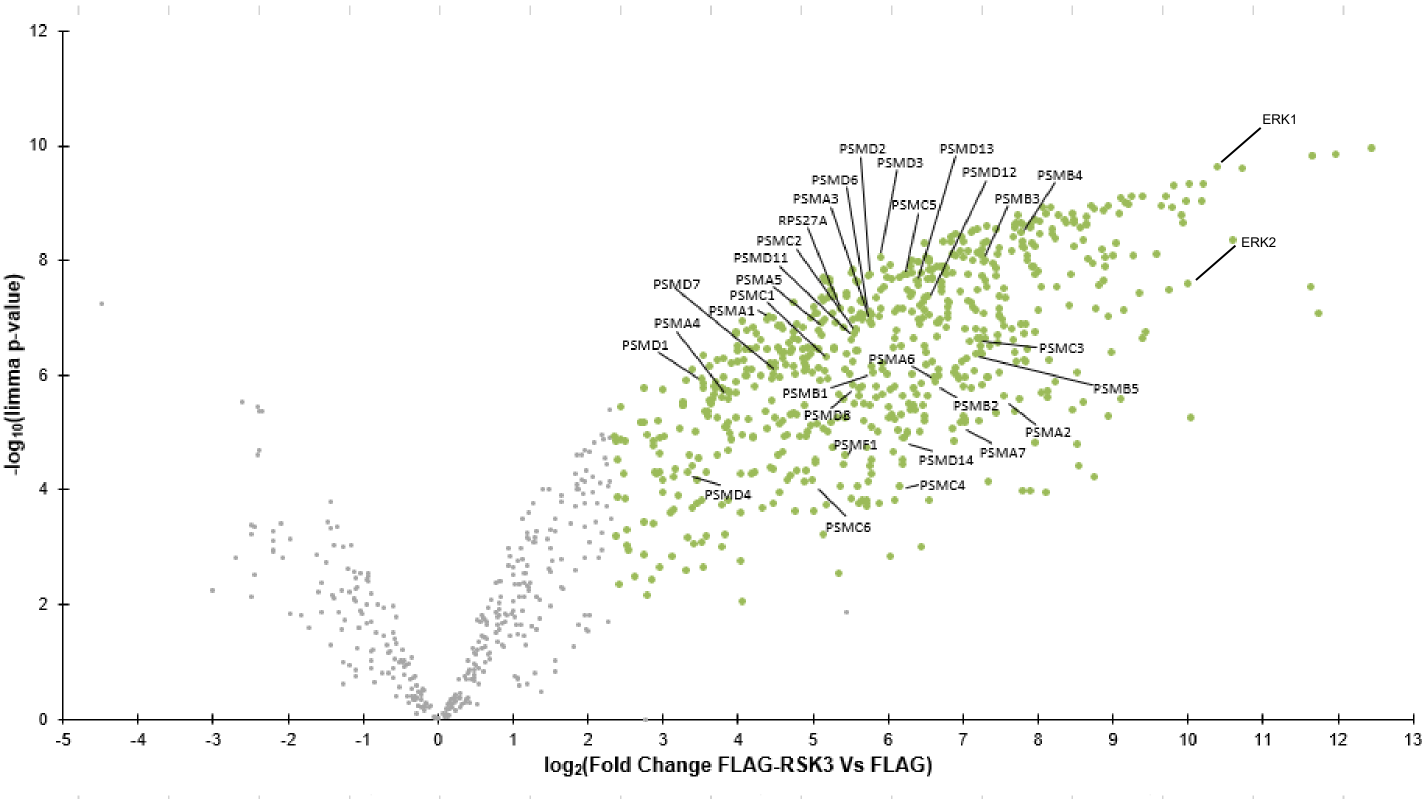

B

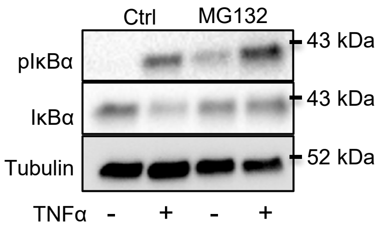

Figure S4. (A) MS-based proteomic characterization of RSK3 interactome. Volcano plot displaying the differential abundance of proteins in FLAG-RSK3 and FLAG co-IP eluates analyzed by MS-based label-free quantitative proteomics. The volcano plot represents the -log10 (limma p-value) on y axis plotted against the log2(Fold Change FLAG-RSK3/FLAG) on x axis for each quantified protein. Green dots represent proteins found significantly enriched in FLAG-RSK3 eluates (Fold change ≥ 5 and p-value ≤ 0.01, leading to a Benjamini-Hochberg FDR < 1 %). (B) Western blot characterization of pIκBα and IκBα levels. Cells were treated with 40μM MG132 2 h prior stimulation with TNFα for 5 min.

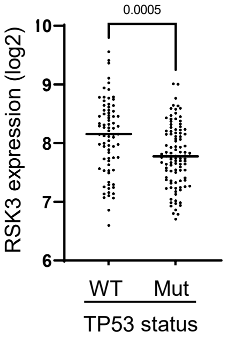

Figure S5. RSK3 expression in TP53 wild-type (WT) and mutated (Mut) Claudin-low breast tumors. Uunpaired t-test was used to determine the statistical significance.

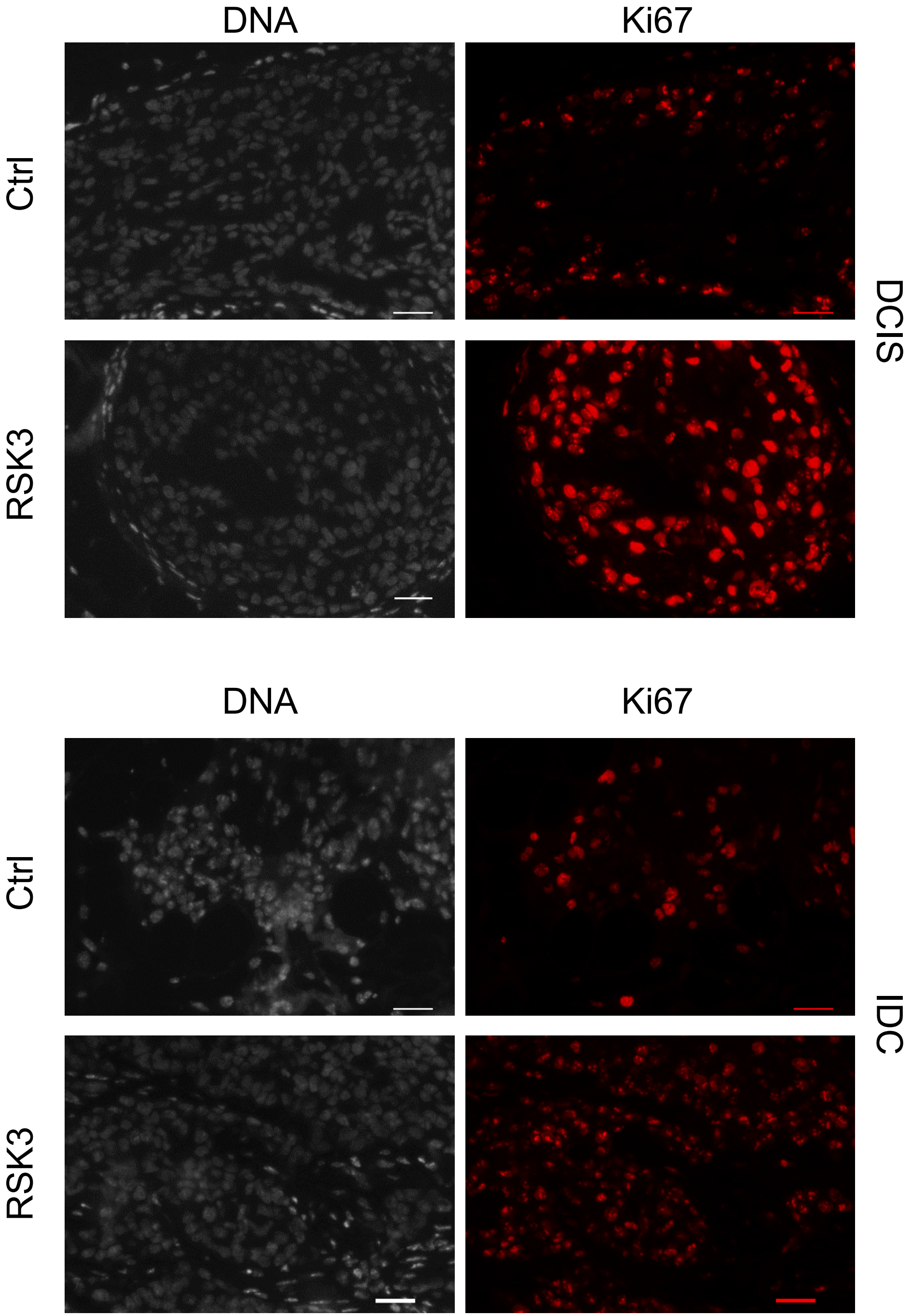

Figure S6. Immunofluorescence against Ki67 proliferation marker on xenografted tumor, overexpressing or not RSK3. DCIS (Ductal Carcinoma in Situ), IDC (Invasive ductal carcinoma). Representative images. Marker size=25µM.

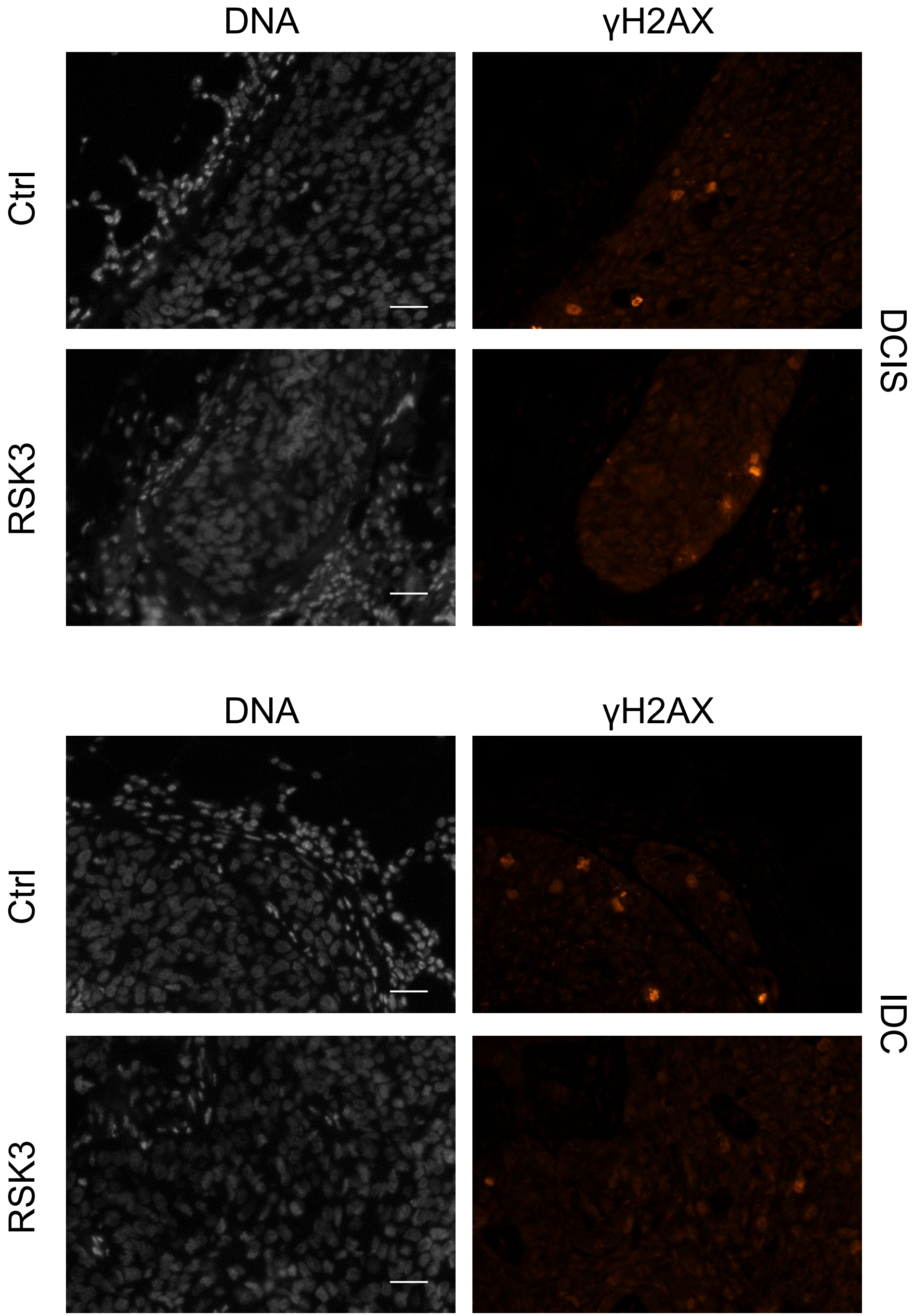

Figure S7. Immunofluorescence against γH2AX DNA damage marker on xenografted tumor, overexpressing or not RSK3. DCIS (Ductal Carcinoma in Situ), IDC (Invasive ductal carcinoma). Representative images. Marker size=25μM.

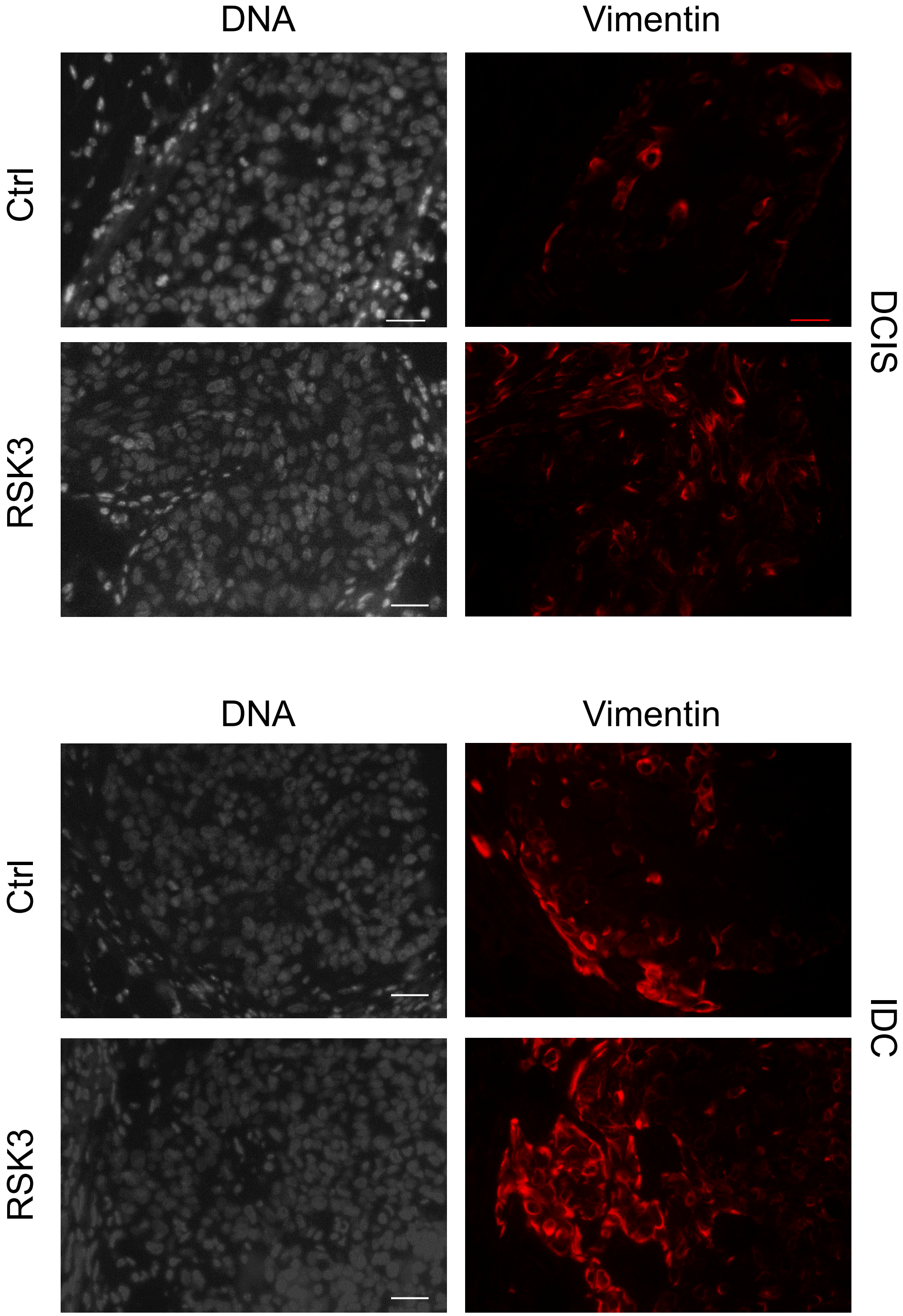

Figure S8. Immunofluorescence against vimentin EMT marker on xenografted tumor, overexpressing or not RSK3. DCIS (Ductal Carcinoma in Situ), IDC (Invasive ductal carcinoma). Representative images. Marker size=25μM.

gene	foreward primer sequence	reverse primer sequence
CDKN1A	TCACTGTCTTGTACCCTTGTGC	GGCGTTTGGAGTGGTAGAAAT
CSF2	TCTCAGAAATGTTTGACCTCCA	CCCTTGAGCTTGGTGAGG
CXCL8	AGACAGCAGAGCACACAAGC	ATGGTTCCTTCCGGTGGT
GAPDH	AGCCACATCGCTCAGACAC	GCCCAATACGACCAAATCC
ICAM1	CCTTCCTCACCGTGTACTGG	AGCGTAGGGTAAGGTTCTTGC
IL1a	GGTTGAGTTTAAGCCAATCCA	TGCTGACCTAGGCTTGATGA
IL6	GATGAGTACAAAAGTCCTGATCCA	CTGCAGCCACTGGTTCTGT
MKI67	TCAAGGAACTGATTCAGGAGAAG	GTGCACTGAAGAACACATTTCC
RSK3	CAGCTCAGCCGACAGGAC	CCAGGTTGGATGACAGCAC
SMAD2	CAGTTGAATCAAAGTATGGACACA	CAAGCTATGATTAACAGGGGAAA
SMAD3	CGATGTCCCCAGCACATAAT	CGATGTCCCCAGCACATAAT
SMAD4	CCCAGGATCAGTAGGTGGAA	GCATAAGCGACGAAGGTCAT

Table S1. List of primers used for RT-QPCR.

Primary antibodies

protein	antibody	host	concentration	producer	cat nr
E-cadherin	Purified mouse Anti E-cadherin	mouse	WB 1/1000, IF 1/500	BD Biosciences	610181
Fibronectin	Mouse Monoclonal antibody to Fibronectin (EP5)	mouse	WB 1/500, IF 1/200	Santa Cruz Biotechnology	sc-8422
FLAG	Monoclonal ANTI-FLAG® M2 antibody	mouse	WB 1/1000	Sigma-Aldrich	F1804
IκBα	IκBα (L35A5) Mouse mAb (Amino-terminal Antigen)	mouse	WB 1/1000	Cell Signaling Technology	# 4814S
Ki67	Recombinant Anti-Ki67 antibody [SP6]	rabbit	IF 1/500 (tumor)	AbCam	ab11174
pIκBα	Phospho-IκBα (Ser32) (14D4) Rabbit mAb	rabbit	WB 1/1000	Cell Signaling Technology	# 2859S
pγH2AX	Rabbit polyclonal to gamma H2A.X (phospho S139)	rabbit	IF 1/500 (tumor)	AbCam	ab16667
PSMB4	Anti-PSMB4 Antibody (H-3)	mouse	WB 1/500	Santa Cruz Biotechnology	sc-390878
PSMB5	Anti-20S Proteasome β5 Antibody (A-10)	mouse	WB 1/500	Santa Cruz Biotechnology	sc-393931
RSK3	Rsk-3 (3C4C8) Antibody	mouse	WB 1/500, detects endogenous protein	Santa Cruz Biotechnology	sc-517283
RSK3	RSK3 Antibody	rabbit	WB 1/500, detects overexpression only	Cell Signaling Technology	9343S
SMAD3	SMAD3	rabbit	WB 1/1000	Abcam	ab28379
Tubulin	Anti-alpha Tubulin antibody [DM1A]	mouse	WB 1/5000	Abcam	ab7291
Vimentin	Anti-Vimentin monoclonal mouse clone V9	mouse	WB 1/500, IF 1/200	Dako	MO725
Vimentin	Rabbit polyclonal to Vimentin	rabbit	IF 1/500 (tumor)	AbCam	ab137321

Secondary antibodies

	Goat anti-Mouse IgG (H+L) Secondary Antibody, Alexa Fluor™ 488	goat	IF 1/500	Invitrogen	A-11001
	Goat Anti-Rabbit IgG H&L (Alexa Fluor® 568)	goat	IF 1/500	AbCam	ab175471
	Peroxidase AffiniPure Donkey Anti-Mouse IgG (H+L)	donkey	WB 1/5000	Jackson Immunoresearch	715-035-150
	Peroxidase AffiniPure Donkey Anti-Rabbit IgG (H+L)	donkey	WB 1/5000	Jackson Immunoresearch	711-035-152

Table S2. List of antibodies used.

kinase vector	FC (log2)	kinase vector	FC (log2)
pWZLneo/ADCK4	-1,1595	pWZLneo/AKT1	-0,0828
pWZLneo/EPHA4	-1,0932	pWZLneo/MVK	-0,0773
pWZLneo/PRKCD	-1,0728	pWZLneo/UCK2	-0,0681
pWZLneo/PDK1	-1,0258	pWZLneo/PRKAR2A	-0,0679
pWZLneo/FGFR1	-0,9645	pWZLneo/TESK1	-0,0617
pWZLneo/MAP3K14	-0,9379	pWZLneo/SNF1LK	-0,0584
pWZLneo/STK32A	-0,8957	pWZLneo/GRKHexokinase 4 flag	-0,052
pWZLneo/ADRBK2 flag	-0,8612	pWZLneo/SPHK2	-0,0504
pWZLneo/PKN1	-0,8521	pWZLneo/ITPK1	-0,0502
pWZLneo/MOBKL1A	-0,8431	pWZLneo/DYRK4	-0,045
pWZLneo/RPS6KB1	-0,8196	pWZLneo/MAPK12	-0,0434
pWZLneo/AXL	-0,8102	pWZLneo/CKB	-0,038
pWZLneo/NME7	-0,7838	pWZLneo/ILK	-0,0307
pWZLneo/TTKflag	-0,7801	pWZLneo/PNKP	-0,0267
pWZLneo/TIE1	-0,7548	pWZLneo/SYK	-0,0216
pWZLneo/NTRK3	-0,7482	pWZLneo/RPS6KL1	-0,0072
pWZLneo/MPP1	-0,6969	pWZLneo/YES1	-0,0003
pWZLneo/FGR	-0,6917	pwzl	0
pWZLneo/PKM2	-0,6876	pWZLneo/PRKCZ	0,00019
pWZLneo/STK38L	-0,6862	pWZLneo/PRKRA	0,00144
pWZLneo/CAMKK1	-0,6807	pWZLneo/BTK	0,00644
pWZLneo/PFKM	-0,6392	pWZLneo/PCTK2	0,00839
pWZLneo/TNK2	-0,6364	pWZLneo/NADK	0,01584
pWZLneo/SRPK2	-0,6267	pWZLneo/CDK2	0,02921
pWZLneo/TEC	-0,6196	pWZLneo/PLK2flag	0,03061
pWZLneo/MATK	-0,6147	pWZLneo/CDC2	0,03428
pWZLneo/ITPKB	-0,5843	pWZLneo/GCK	0,03672
pWZLneo/PTK2 (flag)	-0,5712	pWZLneo/PRKAA1	0,04316
pWZLneo/RIOK3	-0,553	pWZLneo/PLK1flag	0,05405
pWZLneo/MAP2K6	-0,5376	pWZLneo/PI4K2B	0,07218
pWZLneo/PFKL	-0,5094	pWZLneo/PIK3R3	0,07744
pWZLneo/CSNK1G1	-0,4859	pWZLneo/CDK7	0,08049
pWZLneo/TSSK6	-0,4812	pWZLneo/BLK	0,09194
pWZLneo/CLK2	-0,4762	pWZLneo/MOBKL2A	0,09897
pWZLneo/DGKG	-0,4542	pWZLneo/CLK3	0,10446
pWZLneo/TBK1	-0,4528	pWZLneo/MAP2K5	0,10513
pWZLneo/CALM2	-0,4352	pWZLneo/MAPK14	0,11853
pWZLneo/PAK4	-0,4333	pWZLneo/NEK3	0,11853
pWZLneo/PDXK	-0,4302	pWZLneo/NEK6flag	0,12772
pWZLneo/RPS6KA6	-0,4302	pWZLneo/STK32B	0,12837
pWZLneo/RET	-0,428	pWZLneo/MAPK6	0,13001
pWZLneo/CKMT2	-0,4218	pWZLneo/RPS6KA5	0,1305
pWZLneo/PMVK	-0,4137	pWZLneo/CKM	0,13864
pWZLneo/CKMT1A	-0,4094	pWZLneo/ITK	0,14302
pWZLneo/CSNK1E	-0,4056	pWZLneo/DAK	0,1435
pWZLneo/MAP3K8	-0,3945	pWZLneo/CMPK	0,1456
pWZLneo/STK40	-0,3798	pWZLneo/MAPK7	0,1535
pWZLneo/FRK	-0,3707	pWZLneo/CAMK2B	0,1543
pWZLneo/TYK2	-0,3636	pWZLneo/ADCK5	0,15479
pWZLneo/DLG5	-0,3526	pWZLneo/GRK6	0,15959
pWZLneo/MAPKAP1	-0,3524	pWZLneo/LIMK1	0,16167
pWZLneo/IHPK2	-0,3446	pWZLneo/RPS6KB2	0,16646
pWZLneo/MAPK13	-0,3428	pWZLneo/CKS2	0,17408
pWZLneo/GRK5	-0,3419	pWZLneo/MAST1	0,19781
pWZLneo/MAP3K6	-0,3288	pWZLneo/TAOK3	0,20139
pWZLneo/PIK4CB	-0,3279	pWZLneo/AMHR2	0,20558
pWZLneo/PIK4CA	-0,325	pWZLneo/CDK9	0,20992
pWZLneo/PIK3CB	-0,3185	pWZLneo/PCTK3	0,21301
pWZLneo/PLK4flag	-0,313	pWZLneo/PIK3CG	0,21701
pWZLneo/HCK	-0,3039	pWZLneo/STK33	0,21716
pWZLneo/AKT3	-0,3008	pWZLneo/MELK	0,2207
pWZLneo/BMX	-0,2995	pWZLneo/PKN2	0,22407
pWZLneo/CAMK1G	-0,296	pWZLneo/PIP5K3	0,22605
pWZLneo/CAMK2D	-0,2896	pWZLneo/MAP2K7	0,23292
pWZLneo/Hexokinase 2 flag	-0,2668	pWZLneo/VRK2	0,23656
pWZLneo/GAKflag	-0,2651	pWZLneo/PIM1	0,24322
pWZLneo/PDPK1	-0,2566	pWZLneo/GK2	0,24985
pWZLneo/STK17B	-0,2502	pWZLneo/CKS1B	0,2521
pWZLneo/CERK	-0,2493	pWZLneo/LCK	0,256
pWZLneo/STK4	-0,2447	pWZLneo/PBK	0,26525
pWZLneo/PCTK1	-0,236	pWZLneo/STK32C	0,26867
pWZLneo/IKBKE	-0,2345	pWZLneo/GALK2	0,27356
pWZLneo/PLK3flag	-0,2318	pWZLneo/AURORA A flag	0,27858
pWZLneo/NEK11	-0,2222	pWZLneo/PIP5K1A	0,28123
pWZLneo/TSSK1B	-0,2141	pWZLneo/PRKACG flag	0,28519
pWZLneo/ULK4	-0,2081	pWZLneo/MAP3K7	0,30934
pWZLneo/RIOK1	-0,2066	pWZLneo/CLK1	0,31136
pWZLneo/Hexokinase 1 flag	-0,2	pWZLneo/ADPGK	0,32284
pWZLneo/NUAK2	-0,2	pWZLneo/ADRBK1	0,32541
pWZLneo/PRKACB flag	-0,1955	pWZLneo/CSNK1A1L	0,32598
pWZLneo/AAK1	-0,178	pWZLneo/PRKAG2	0,32826
pWZLneo/CDK5	-0,1694	pWZLneo/PCK2	0,34454
pWZLneo/PIK3R5	-0,1573	pWZLneo/PLAU	0,35785
pWZLneo/HIPK1	-0,1548	pWZLneo/DYRK2	0,36272
pWZLneo/PDIK1L	-0,1427	pWZLneo/STK3	0,3673
pWZLneo/Hexokinase 3 flag	-0,1368	pWZLneo/RIOK2	0,37339
pWZLneo/PACSIN1	-0,1346	pWZLneo/OXSR1	0,38425
pWZLneo/CAMK4	-0,1326	pWZLneo/RPS6KA2	0,38862
pWZLneo/PRKCI	-0,1281	pWZLneo/PAPSS1	0,42689
pWZLneo/CAMKV	-0,1229	pWZLneo/CDK4	0,45559
pWZLneo/DGUOK	-0,1184	pWZLneo/CSNK1G2	0,46287
pWZLneo/PIP5K2A	-0,1159	pWZLneo/CHEK1	0,49417
pWZLneo/LIMK2	-0,1033	pWZLneo/TK1	0,49861
pWZLneo/RPSK6A3	-0,1014	pWZLneo/SGK	0,52108
pWZLneo/FASTK	-0,0978	pWZLneo/ACVR1	0,5848
pWZLneo/VRK3	-0,0862	pWZLneo/PIP5K1B	0,62453
pWZLneo/MKNK1	-0,0833		

Table S3. Results of the genetic screen of HMECT cells infected with activated kinases and treated with TGFβ. Results are displayed as FC(log2) comparing cell counts with a given kinase relative to pwzl control vector.

Transcription Factor	Direction of regulated genes		Q.value
NFKB1	TGFB UP	RSK3 DOWN	0
RELA	TGFB UP	RSK3 DOWN	0
E2F1	TGFB DOWN	RSK3 UP	0,00276
JUN	TGFB UP	RSK3 DOWN	0,00483
BRCA1	TGFB UP	RSK3 DOWN	0,00483

Table S4. Results of TFacts analysis. Common genes: up-regulated in HMECT-pWZL treated with TGFβ compared to non-treated control (TGFβ UP) and down-regulated in HMECT-pWZL/RSK3 treated with TGFβ compared to HMECT-pWZL treated with TGFβ (RSK3 DOWN), or the opposite were used for identification of transcription factors.

Annotation Cluster 2	Enrichment Score: 17.64		
		P_Value	Benjamini
UP_KEYWORDS	Proteasome	5.70E-34	7.20E-32
GOTERM_BP_DIRECT	positive regulation of ubiquitin-protein ligase activity involved in regulation of mitotic cell cycle transition	8.20E-34	1.10E-30
GOTERM_BP_DIRECT	negative regulation of ubiquitin-protein ligase activity involved in mitotic cell cycle	9.30E-34	1.10E-30
GOTERM_BP_DIRECT	anaphase-promoting complex-dependent catabolic process	5.10E-33	4.20E-30
GOTERM_CC_DIRECT	proteasome complex	6.90E-32	9.60E-30
GOTERM_BP_DIRECT	regulation of mRNA stability	2.20E-30	1.30E-27
GOTERM_BP_DIRECT	regulation of cellular amino acid metabolic process	2.30E-29	1.10E-26
GOTERM_BP_DIRECT	antigen processing and presentation of exogenous peptide antigen via MHC class I, TAP-dependent	1.10E-28	4.30E-26
GOTERM_BP_DIRECT	NIK/NF-kappaB signaling	6.70E-28	2.40E-25
KEGG_PATHWAY	Proteasome	1.70E-27	4.00E-25
GOTERM_BP_DIRECT	stimulatory C-type lectin receptor signaling pathway	6.80E-23	1.80E-20
GOTERM_BP_DIRECT	proteasome-mediated ubiquitin-dependent protein catabolic process	7.70E-23	1.90E-20
GOTERM_BP_DIRECT	Wnt signaling pathway, planar cell polarity pathway	1.30E-22	2.90E-20
GOTERM_CC_DIRECT	proteasome accessory complex	6.20E-21	5.80E-19
GOTERM_BP_DIRECT	positive regulation of canonical Wnt signaling pathway	8.00E-20	1.60E-17
GOTERM_BP_DIRECT	protein polyubiquitination	4.00E-19	7.60E-17
GOTERM_BP_DIRECT	T cell receptor signaling pathway	9.10E-19	1.60E-16
GOTERM_BP_DIRECT	tumor necrosis factor-mediated signaling pathway	4.90E-18	8.10E-16
GOTERM_BP_DIRECT	Fc-epsilon receptor signaling pathway	7.10E-18	1.10E-15
GOTERM_BP_DIRECT	negative regulation of canonical Wnt signaling pathway	2.20E-17	3.20E-15
GOTERM_BP_DIRECT	MAPK cascade	1.70E-14	2.20E-12
GOTERM_CC_DIRECT	proteasome core complex	3.30E-13	2.00E-11
UP_KEYWORDS	Threonine protease	2.00E-12	4.90E-11
INTERPRO	Proteasome, subunit alpha/beta	2.40E-12	9.00E-10
GOTERM_MF_DIRECT	threonine-type endopeptidase activity	2.10E-11	3.30E-09
KEGG_PATHWAY	Epstein-Barr virus infection	3.60E-10	4.30E-08
INTERPRO	Proteasome A-type subunit	3.10E-08	2.50E-06
INTERPRO	Proteasome, alpha-subunit, N-terminal domain	3.10E-08	2.50E-06
GOTERM_CC_DIRECT	proteasome core complex, alpha-subunit complex	3.30E-08	9.20E-07
SMART	SM00948	3.70E-08	4.00E-06
GOTERM_BP_DIRECT	ubiquitin-dependent protein catabolic process	4.20E-07	2.60E-05
GOTERM_BP_DIRECT	proteolysis involved in cellular protein catabolic process	6.10E-07	3.60E-05
INTERPRO	Proteasome, beta-type subunit, conserved site	2.40E-05	9.20E-04
INTERPRO	Proteasome B-type subunit	3.10E-04	8.90E-03

Table S5. Pathway analysis of RSK3 binding proteins. Analysis was performed with DAVID online software.

Gene name	FC (RSK3 vs empty)	p value
PSMB4	220.4	4.66E-09
PSMB3	151.9	8.29E-09
PSMA2	186.8	2.33E-06
PSMC3	151.2	3.00E-07
PSMB5	143.7	4.11E-07
PSMD12	92.6	4.26E-08
PSMA7	130.8	6.74E-06
PSMD13	83.0	2.27E-08
PSMB2	99.6	1.34E-06
PSMA6	97.2	1.11E-06
PSMC5	74.3	1.77E-08
PSMD3	59.4	8.46E-09
PSMD2	53.6	1.84E-08
PSMA3	54.8	8.99E-08
PSMD6	54.1	7.68E-08
PSMD14	73.7	1.23E-05
PSMB1	55.7	8.69E-07
PSMC2	47.6	1.52E-07
PSMD11	46.8	1.85E-07
RPS27A	41.2	6.67E-08
PSMC4	70.7	8.38E-05
PSMD8	46.4	1.47E-06
PSMA5	34.6	1.22E-07
PSMC1	36.2	4.72E-07
PSMF1	43.1	2.47E-05
PSMA1	21.3	9.50E-08
PSMD7	22.4	7.35E-07
PSMC6	31.9	6.88E-05
PSMA4	14.1	1.82E-06
PSMD1	11.4	1.15E-06
PSMD4	10.0	4.76E-05
SKP1	8.5	2.54E-04

Table S6. List of proteasome components ($p < 1E-04$) identified by mass spectrometry on FLAG-RSK3 immunoprecipitates.

Epithelial

Gene	Correlation coefficient	p value
ADGRG1_9289_EMT_PANCANCER_DN	-0.05758041	0.01197272
AP1G1_164_EMT_PANCANCER_DN	-0.17256953	3.4014E-14
ATP8B1_5205_EMT_PANCANCER_DN	-0.17216593	3.9072E-14
CDS1_1040_EMT_PANCANCER_DN	-0.06077988	0.00798196
CGN_57530_EMT_PANCANCER_DN	-0.14720114	1.0889E-10
CLDN4_1364_EMT_PANCANCER_DN	-0.13127972	8.9504E-09
CNOT1_23019_EMT_PANCANCER_DN	0.00074209	0.97418525
CTNND1_1500_EMT_PANCANCER_DN	0.10179149	8.5749E-06
DYNC1LI2_1783_EMT_PANCANCER_DN	0.27700788	6.9694E-35
ERBB3_2065_EMT_PANCANCER_DN	-0.11888434	1.9657E-07
ESRP1_54845_EMT_PANCANCER_DN	-0.307854	4.4429E-43
ESRP2_80004_EMT_PANCANCER_DN	-0.07460635	0.00112264
F11R_50848_EMT_PANCANCER_DN	-0.20965777	2.3455E-20
GRHL2_79977_EMT_PANCANCER_DN	-0.18471991	4.4756E-16
HOOK1_51361_EMT_PANCANCER_DN	-0.14736022	1.0393E-10
IRF6_3664_EMT_PANCANCER_DN	-0.08786538	0.00012359
MAP7_9053_EMT_PANCANCER_DN	-0.07605614	0.0008959
MARVELD2_153562_EMT_PANCANCER_DN	-0.2156275	1.82E-21
MARVELD3_91862_EMT_PANCANCER_DN	-0.08322852	0.00027735
MYO5B_4645_EMT_PANCANCER_DN	-0.10229521	7.7332E-06
OCLN_100506658_EMT_PANCANCER_DN	-0.15581682	8.1224E-12
PRSS8_5652_EMT_PANCANCER_DN	0.03184624	0.16481891
SPINT1_6692_EMT_PANCANCER_DN	-0.06776846	0.00309114

Mesenchymal

Gene	Correlation coefficient	p value
ADAM12_8038_EMT_PANCANCER_UP	0.13524148	3.1305E-09
ADAMTS12_81792_EMT_PANCANCER_UP	-0.00108529	0.96225405
ADAMTS2_9509_EMT_PANCANCER_UP	0.3795252	2.7877E-66
AEBP1_165_EMT_PANCANCER_UP	0.43112619	4.9722E-87
ANGPTL2_23452_EMT_PANCANCER_UP	0.4685644	1.62E-104
ANTXR1_84168_EMT_PANCANCER_UP	0.29228625	8.1436E-39
AXL_558_EMT_PANCANCER_UP	0.4075968	4.101E-77
BNC2_54796_EMT_PANCANCER_UP	0.39169965	7.5187E-71
CALD1_800_EMT_PANCANCER_UP	0.33755153	5.8767E-52
CDH2_1000_EMT_PANCANCER_UP	0.13027708	1.162E-08
CMTM3_123920_EMT_PANCANCER_UP	0.44623617	7.9612E-94
CNRIP1_25927_EMT_PANCANCER_UP	0.3938534	1.1157E-71
COL10A1_1300_EMT_PANCANCER_UP	0.17840619	4.4127E-15
COL1A1_1277_EMT_PANCANCER_UP	0.40433089	8.4656E-76
COL1A2_1278_EMT_PANCANCER_UP	0.35434682	1.9758E-57
COL3A1_1281_EMT_PANCANCER_UP	0.34235788	1.7258E-53
COL5A1_1289_EMT_PANCANCER_UP	0.44164885	1.0014E-91
COL5A2_1290_EMT_PANCANCER_UP	0.37884866	4.9375E-66
COL6A1_1291_EMT_PANCANCER_UP	0.45038407	9.4311E-96
COL6A2_1292_EMT_PANCANCER_UP	0.48507864	5.957E-113
COL6A3_1293_EMT_PANCANCER_UP	0.39044549	2.2689E-70
COL8A1_1295_EMT_PANCANCER_UP	0.35136385	1.9609E-56
DACT1_51339_EMT_PANCANCER_UP	0.32277347	2.0518E-47
EMP3_2014_EMT_PANCANCER_UP	0.22885476	4.7888E-24
FAP_2191_EMT_PANCANCER_UP	0.36065244	1.4229E-59
FBN1_2200_EMT_PANCANCER_UP	0.40337082	2.0482E-75
FN1_2335_EMT_PANCANCER_UP	0.16353311	6.9998E-13
FSTL1_11167_EMT_PANCANCER_UP	0.45508936	5.7113E-98
GPC6_10082_EMT_PANCANCER_UP	0.17157254	4.7876E-14
GYPC_2995_EMT_PANCANCER_UP	0.16021453	2.0386E-12
HTRA1_5654_EMT_PANCANCER_UP	0.49055958	7.446E-116
INHBA_3624_EMT_PANCANCER_UP	-0.04723998	0.03929288
ITGA11_22801_EMT_PANCANCER_UP	0.31362534	9.999E-45
LOXL2_4017_EMT_PANCANCER_UP	-0.00885483	0.69939814
LRRC15_131578_EMT_PANCANCER_UP	0.30809344	3.8022E-43
MMP2_4313_EMT_PANCANCER_UP	0.44403008	8.2172E-93
MSRB3_253827_EMT_PANCANCER_UP	0.47739992	5.681E-109
NAP1L3_4675_EMT_PANCANCER_UP	0.26848802	8.5191E-33
NID2_22795_EMT_PANCANCER_UP	0.10982937	1.557E-06
OLFML2B_25903_EMT_PANCANCER_UP	0.41917621	6.7855E-82
PCOLCE_5118_EMT_PANCANCER_UP	0.48494648	6.989E-113
PDGFRB_5159_EMT_PANCANCER_UP	0.52766738	5.761E-137
PMP22_5376_EMT_PANCANCER_UP	0.02757489	0.22910612
POSTN_10631_EMT_PANCANCER_UP	0.1848922	4.1998E-16
SPOCK1_6695_EMT_PANCANCER_UP	0.37181693	1.7345E-63
SULF1_23213_EMT_PANCANCER_UP	0.16863916	1.2937E-13
SYT11_23208_EMT_PANCANCER_UP	0.31205886	2.8237E-44
THBS2_7058_EMT_PANCANCER_UP	0.41878817	9.8826E-82
VCAN_1462_EMT_PANCANCER_UP	0.35215296	1.0711E-56
VIM_7431_EMT_PANCANCER_UP	0.26726379	1.6756E-32
ZEB2_9839_EMT_PANCANCER_UP	0.18027272	2.2627E-15

Table S7. Analysis of the METABRIC breast cancer database. Correlation coefficients between expression of indicated epithelial or mesenchymal factors and RSK3. p-values were determined by linear regression analysis, green means negative correlation and red positive correlation.
